# Intersecting Epidemics: A Multilevel Syndemic Analysis of a Chikungunya Virus Epidemic in Colombia Through Clinical, Biological, and Socioeconomic Factors

**DOI:** 10.3390/v18050549

**Published:** 2026-05-09

**Authors:** Juan C. Rueda, Ana María Santos, Ignacio Angarita, Ingris Peláez-Ballesta, Alfonso Gastelum, Igor Rueda, Jaime Cortés-Ramos, Cristian Astudillo, Daniel Rincón-Sierra, Karina Guzmán, Jesús Giovanny Ballesteros, Juan Manuel Bello, John Londono

**Affiliations:** 1 Spondyloarthritis Study Group (GESPA), Universidad de La Sabana, Km 7 Autopista Norte, Chía 250001, Colombia; juan.rueda@unisabana.edu.co (J.C.R.); ana.santos@unisabana.edu.co (A.M.S.); ignacio.angarita@unisabana.edu.co (I.A.); igorruca@unisabana.edu.co (I.R.); jaimecora@unisabana.edu.co (J.C.-R.); cristianasbu@unisabana.edu.co (C.A.); danielrisi@unisabana.edu.co (D.R.-S.); karinaguro@unisabana.edu.co (K.G.); 2 Bioscience Programme, Faculty of Medicine and Engineering, Universidad de La Sabana, Km 7 Autopista Norte, Chía 250001, Colombia; 3 Rheumatology Department, Hospital General de México Dr. Eduardo Liceaga, Dr. Balmis No. 148, Col. Doctores, Ciudad de México 06720, Mexico; pelaezin@gmail.com; 4 Instituto de Ciencias Aplicadas y Tecnología (ICAT), Universidad Nacional Autónoma de México, Ciudad Universitaria, Ciudad de México 04510, Mexico; alfonso.gastelum@icat.unam.mx; 5 Rheumatology Department, Hospital Militar Central, Transversal 9 No. 136-21, Bogotá 110121, Colombia; giovannyballesteros@yahoo.com (J.G.B.); juan.bello@unimilitar.edu.co (J.M.B.)

**Keywords:** chikungunya virus (CHIKV), syndemic, cytokine profiling, immune response, multi-level analysis, socioeconomic status

## Abstract

This study applied a syndemic framework to chikungunya virus (CHIKV) infection during the 2014–2015 Colombian epidemic, integrating biological and social determinants. Methods: A community-based cohort of 279 serologically confirmed adults from six cities was analyzed. Clinical, sociodemographic, and cytokine data were evaluated using multilevel and multivariate statistical approaches. Results: Among 279 patients, 141 (50.5%) met World Health Organization (WHO) criteria for acute CHIKV infection. The cohort was predominantly female and of lower socioeconomic status (SES). The most frequent manifestations were arthralgia (91%), fatigue (58%), fever (50.5%), myalgia (45.9%), and rash (45.2%). Multivariate models identified IL-15, IL-17A, IL-12p40, MCP-1, and MIP-1α as significant correlates of fever, rash, and myalgia. Socioeconomic and ethnic factors influenced cytokine expression; Caucasian patients showed higher proinflammatory cytokine levels than Afro-American patients. Lower SES was associated with greater symptom burden. Network analyses revealed distinct immune signatures linking biological responses with clinical and demographic variables. Conclusion: Immune responses, clinical manifestations, and social disadvantages interact significantly in CHIKV infection. These findings support a syndemic model in which socioeconomic vulnerability amplifies disease impact, highlighting the need for integrated biosociological public health strategies, particularly targeting populations with low socioeconomic status.

## 1. Introduction

The term “syndemic” was first described in 1996 by Singer, who coined the term to analyze how two or more diseases cluster together; how social, economic, and ecological factors influence these clusters; and how the clustered conditions interact through biological, psychological, or social pathways [1]. The main goal of syndemics is to join distinct features of medical theories and social science to formulate predictions for clinical intervention and public health program application [2]. The way the theory of syndemics addresses disease interaction is by stating that co-occurring epidemics interact at the level of populations and individuals with mutually enhancing deleterious consequences for health, in which they can co-occur independently, co-occur and are mutually enhancing (interacting with each other), or co-occur and are mutually causal (cause each other) [3,4]. This complexity involves multiple levels of analysis to properly prove interaction. It is known that clinical expression of infectious disease is understood as a product of an intricate interplay between an infectious agent, the host’s immune response, and environmental factors [5]. That is why multiple studies on HIV infection or HIV transmission risk behaviors as models for syndemic interaction theory have been published. However, this syndemic analytical framework has not yet been applied in systematic, multilevel empirical research on the population affected by the CHIKV in Colombia.

During 2014 and 2015, a CHIKV epidemic took place in Colombia. CHIKV is an alphavirus from the *Togaviridae* family, and a member of the Semliki Forest virus antigenic complex, that together with other alphaviruses (*O’nyong-nyong*, *Mayaro*, and Ross River) causes acute arthropathy in humans [6,7,8]. Studies of CHIKV-infected humans and animals have defined symptoms and immune responses of acute CHIKV disease, but much of the molecular interplay between virus and host remains to be established. To this date, it is unknown why a percentage of patients do not develop clinical symptoms despite having been exposed to the virus and despite developing an adaptive immune response (presence of positive CHIKV IgM or IgG).

The clinical manifestation of infectious diseases is often recognized as a result of a complex interaction involving the infectious agent, the host’s immune response, and many environmental factors. The theoretical approach to study those interactions is called syndemics. To study those interactions, we hypothesize that biomolecular associations represented in our study by cytokine interaction, as well as bio-clinical interplay represented by cytokine and clinical symptoms, and finally environmental factors represented by ecological, social, and economic variables, cooperate to explain the complexity of an infectious disease within a syndemic framework. To investigate the syndemics of CHIKV in a Colombian cohort, a multilevel analysis was made.

The use of syndemics theory will provide an approach for identifying the conditions that cause or exacerbate CHIKV infection, as well as targeting underlying patterns of multidirectional disease causality to design better clinical interventions.

From a rheumatology perspective, CHIKV is clinically important not only as an acute infection but as a trigger of persistent musculoskeletal morbidity—polyarthralgia, inflammatory arthritis, tenosynovitis, fatigue, and functional disability—which often resemble or overlap with chronic rheumatic diseases, particularly rheumatoid arthritis, spondyloarthritis, and undifferentiated polyarthritis.

A syndemics framework is useful because it explains this heterogeneity as the result of interacting layers rather than a single exposure. In our approach, biomolecular networks (cytokine–cytokine interactions) and bioclinical links (cytokines associated with symptom clusters) are examined together to clarify why some seropositive individuals remain asymptomatic while others progress to chronic, disabling phenotypes [7,8].

Syndemics theory goes beyond describing comorbidity by testing whether co-occurring conditions and contextual factors are mutually reinforcing—biologically, psychologically, and socially. This is particularly relevant in CHIKV because inflammatory pathways, pain processing, and mental health factors can amplify one another and shape long-term outcomes. Identifying distinct post-CHIKV phenotypes (e.g., inflammation-predominant vs. pain/fatigue-predominant patterns) can support clinically actionable stratification and guide integrated management, combining anti-inflammatory strategies when appropriate with rehabilitation and multidisciplinary approaches for chronic pain and functional recovery [7,8].

From a global health perspective, CHIKV is a model of how infectious diseases translate into long-term disability and inequality. Transmission and outcomes are shaped by socio-ecological determinants (climate variability, urbanization, housing and water storage, poverty, informal work, gender roles) and by health-system capacity (access, diagnosis, continuity, rehabilitation and mental health services). A multilevel syndemics analysis in a Colombian cohort therefore provides internationally relevant evidence by linking eco-social vulnerability to immune and symptom trajectories, and by informing intervention packages that address multidirectional causality—vector control plus equitable access to early care, follow-up, rehabilitation, and psychosocial support to reduce persistent rheumatic disability [7,8].

This study aims to reveal the syndemic characteristics of CHIKV infection in Colombia and clarify the interaction mechanism of bio-social factors by integrating cytokine profiling, clinical symptoms, and sociodemographic factors.

## 2. Materials and Methods

### 2.1. Study Population

This was an ecological multilevel cluster network analysis nested in a community cohort, including patients aged >18 years, from six Colombian cities between 2014 and 2015, that used the Community Oriented Program for Control of Rheumatic Diseases (COPCORD) stratified sampling method to include patients. A multistage random sampling method was used: primary unit—city; secondary unit—city blocks within each city; and tertiary unit—households. The COPCORD strategy consists of three phases: in the first phase, patients with musculoskeletal symptoms of non-traumatic origin are identified through a direct interview; in the second phase, symptoms are classified based on pain or functional limitation using questionnaires; and, finally, with the involvement of a specialist physician, a rheumatological diagnosis is established. Its application makes it possible to determine the prevalence of these diseases, their distribution, and potential intervention factors as public health strategies. Using this strategy, we conducted the study and collected data on the Colombian population; this methodology is described in greater detail in the publication by Londono et al. (2018) [9,10]. If a patient was positive for COPCORD, interviewers were ordered to ask about CHIKV-related symptoms like fever, rash, myalgia, and fatigue, as well as possible diagnosis of CHIKV infection by a health care worker or by the patient. If positive, CHIKV infection was suspected, a follow-up examination was conducted in the next seven days by a trained rheumatologist or a rheumatology fellow, during which chikungunya fever was confirmed according to World Health Organization (WHO) criteria [11]. Then, a specific questionnaire was administered, including the time of disease onset and further symptoms, such as joint, dermatological, and gastrointestinal manifestations. Blood samples were also taken. Patients were evaluated in a single cross-sectional assessment. Individuals were excluded if the examiner identified or suspected a defined rheumatic disease based on established classification criteria. Rheumatoid arthritis was classified using the 2012 ACR/EULAR criteria; osteoarthritis according to ACR criteria; Gout was classified according to the Mexico 2010 diagnostic criteria; and fibromyalgia using the 2010 ACR criteria.

Spondyloarthritis was classified according to ASAS criteria and ankylosing spondylitis by the modified New York criteria. Systemic lupus erythematosus was defined using the SLICC 2012 criteria. Sjögren’s syndrome was classified according to the 2012 ACR criteria. Idiopathic inflammatory myopathies, including dermatomyositis, were classified using the Bohan and Peter criteria. Systemic sclerosis was defined according to the 2013 ACR/EULAR criteria. Patients with undifferentiated arthritis or spondyloarthritis were also excluded. The definitions of arthralgia and arthritis used were taken from Woolf [12].

In Colombia, residential dwellings receiving public utilities are classified into a six-level socioeconomic stratification system (1–6), where stratum 1 represents the most socioeconomically disadvantaged environments and stratum 6 the most advantaged. This classification is primarily used to differentially assign subsidies and contributions for public services, whereby higher strata (5–6) contribute proportionally more to subsidize lower strata (1–3). Beyond an economic categorization, the system reflects area-level sociodemographic and environmental conditions, as dwellings are classified according to observable indicators linked to living standards and social vulnerability. These include housing construction characteristics, number of habitable rooms and residential density, access to basic water and sanitation services (e.g., piped water supply and indoor sanitation facilities), and neighborhood-level urban development such as paved roads, sewage infrastructure, and availability of public services. Consequently, socioeconomic strata capture structural determinants associated with population living conditions and are frequently used as contextual sociodemographic variables in epidemiological and public health research in Colombia [10,13,14].

The distribution across socioeconomic strata is as follows: strata 1 (*n* = 107, 38.4%), strata 2 (*n* = 99, 35.5%), strata 3 (*n* = 57, 20.4%), strata 4 (*n* = 9, 3.2%), and strata 5 (*n* = 7, 2.5%). No patients were classified in strata 6.

### 2.2. Case Definitions for CHIKV Infection According to World Health Organization Criteria [10]

A case was considered suspect based on clinical criteria (acute onset of fever > 38.5 °C and incapacitating joint pain) and epidemiological criteria (residing in or having visited areas that had reported transmission within 15 days prior to the onset of symptoms). A case was confirmed when the patient met laboratory criteria irrespective of clinical presentation (presence of virus-specific IgM or IgG antibodies in a single serum sample collected in the acute or convalescent stage, respectively). Because our population was immunologically naïve (there were no reports of CHIKV infection prior to this epidemic), we considered the presence of virus-specific IgG antibodies in a single serum sample during any stage of the disease as positive.

### 2.3. CHIKV Serology

Enzyme-linked immunosorbent assay (ELISA) was performed according to the manufacturer’s instructions (Abcam^®^ ab177848 anti-CHIKV IgM human ELISA kit and ab177835 anti-CHIKV IgG human ELISA kit, Abcam, Cambridge, UK). Abcam’s anti-CHIKV IgM Human ELISA kit is reported to produce comparable results to the Centers for Disease Control and Prevention (CDC) IgM ELISA [15,16]. Analytical specifications according to the manufacturer state a specificity and sensitivity > 90% for both IgM and IgG anti-CHIKV.

No cross-reactivity against Bordetella pertussis, Chlamydia trachomatis, Chlamydia pneumoniae, dengue virus (DENV), tick-borne encephalitis (TBE), Helicobacter pylori, herpes simplex virus type 2 (HSV-2), Leishmania, Mycoplasma, or Schistosoma has been reported for IgM. No cross-reactivity against DENV, TBE, cytomegalovirus (CMV), Epstein–Barr virus (EBV), or Helicobacter pylori has been reported for IgG. The manufacturer reports a 10% rate of misclassification with IgG serology.

### 2.4. Cytokine Measurement Method

A multiplex biometric immunoassay, containing fluorescent dyed microspheres conjugated with a monoclonal antibody specific for a target protein, was used for cytokine measurement according to the manufacturer’s instructions (Milliplex^®^ Map Human Cytokine/Chemokine Magnetic Bead Panel, Merck KGaA, Darmstadt, Germany). The following groups of cytokines were analyzed: inflammatory (GM-CSF, IL- 1β, IL-1RA, IL-6, IL-8, TNF-α); Th1/Th2 (IFN-γ, IL-2, IL-4, IL-10); Cytokine II (IFN-α2, IL-7, IL- 12p40/p70, IL-15, IL-17A); chemokines (Eotaxin, IP-10, MCP-1, MIP-1α, MIP-1β); and growth factors (FGF-2) for a total of 21 cytokines analyzed. Detection was performed using the Magpix^®^ System (Luminex Corporation, Austin, TX, USA) with xPONENT^®^ Software version 4.2 for data acquisition. Data analysis was conducted using Milliplex Analyst Software version 5.1 (Luminex Corporation, Austin, TX, USA).

### 2.5. Statistical Analysis

Descriptive analysis was made using means and standard deviation (SD) for continuous variables and count and percentages for categorical variables. Two-by-two tables were used to establish associations between categorical variables. Odds ratios (OR) were calculated for associations with 95% confidence intervals (CI). Student’s *t*-test was used to compare means, and the U Mann–Whitey test to compare continuous and categorical variables when needed; a two-sided *p*-value of <0.05 was considered statistically significant. Positive CHIKV serology (IgG or IgM) was used to identify subjects with CHIKV infection. A bivariate analysis was made, including all studied variables, using suspected CHIKV infection as the outcome. Variables with significant statistical association were included in a multiple regression model using a stepwise backward method. Hosmer and Lemeshow’s goodness-of-fit test was used to assess the model performance. Also, point-biserial and Cramer’s V analyses were performed to evaluate correlations between continuous variables and categorical variables, respectively.

Before multivariate analysis, all variables were standardized to account for differences in measurement scale and variance. Principal component analysis (PCA) was then performed as a dimensionality-reduction method to summarize the underlying correlation structure of the dataset, retaining the minimum number of components needed to explain at least 80% of the total variance [17]. The resulting component scores were subsequently used as input for hierarchical clustering, and the dendrogram was cut at the level showing the most distinct grouping pattern. This PCA–clustering strategy was conducted in three sequential phases: (i) cytokine measurements alone, to identify biological immune-response clusters; (ii) cytokines combined with clinical symptoms, to assess bio-clinical associations; and (iii) cytokines integrated with sociodemographic variables, to explore the interaction between biological and social determinants. This approach reduced the complexity of the dataset while enabling the identification of clinically and biologically meaningful variable clusters within the syndemic framework of CHIKV infection.

Multivariate analyses were performed in SPSS version 22.0 (IBM, Armonk, NY, USA). The first step was to reduce the dimensionality of the dataset by applying a principal-component analysis (PCA) to the full set of measured variables, keeping the minimum number of components to explain at least the 80% of the total variance. The resulting component scores were then subjected to hierarchical clustering, with the dendrogram cut at the level yielding the most distinct separation (the “best hierarchical level”). This PCA–clustering pipeline was executed in three sequential phases:

Phase I (Biological variables only): cytokine concentrations alone were analyzed, revealing three clusters: Cluster 1 (MIP-1, GM-CSF, IL-6, TNF-α), Cluster 2 (IL-15, IL-12, IFN-α, FGF-2) and Cluster 3 (IL-10, IL-8, MCP-1).

Phase II (Cytokines + Clinical symptoms): PCA was repeated on cytokine data combined with binary indicators for arthralgia, fever, fatigue, myalgia, rash and gastrointestinal symptoms, again yielding three clusters each defined by distinct cytokine–symptom signatures.

Phase III (Cytokines + Sociodemographics): integration of cytokine measurements with socioeconomic stratum, ethnicity and education produced four clusters—two predominantly proinflammatory, one anti-inflammatory/chemokine and one mixed cluster—whose composition varied across demographic strata.

### 2.6. Ethical Considerations

This study was carried out according to the Declaration of Helsinki 2013. Broad informed consent was obtained during the 2014–2015 epidemic in the COPCORD study, prior to the patients’ admission. This study corresponds to a retrospective cohort analysis of previously collected data. Ethical approvals were obtained for secondary data analysis from the ethics committees of La Universidad de La Sabana (study approval MED-241-2018, date of approval: 9 November 2018, record No. 450) and Hospital Militar Central (study approval 106-2016, date of approval: 11 November 2016, record No. 20).

## 3. Results

**Figure 1:**
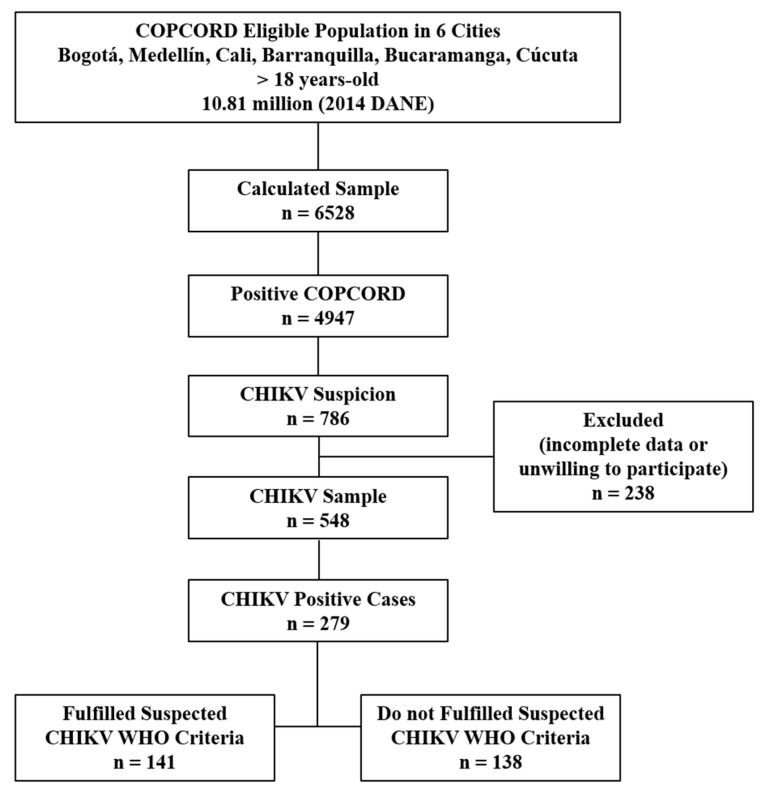
Profile of the study population. DANE: Departamento Administrativo Nacional Estadistico; COPCORD: Community Oriented Program for Control of Rheumatic Diseases; WHO: World Health Organization; CHIKV: Chikungunya Virus.

A total of 279 patients with positive CHIKV serology were analyzed. Of those, 141 (50.5%) fulfilled criteria for suspect case according to WHO (Figure 1 and Figure S1).

### 3.1. Demographics

In general, most patients were female (*n*: 195, 69.9%), hispano (*n*: 133, 47.7%), housewives (*n*: 126, 45.2%), from Barranquilla Colombian Caribbean (*n*: 155, 55.6%), with a mean age of 48.08 years (SD ± 17.6), no income (*n*: 102, 36.6%) and subsidized health insurance (*n*: 162, 58.1%). Table 1.

### 3.2. Clinical Features

**Figure 2:**
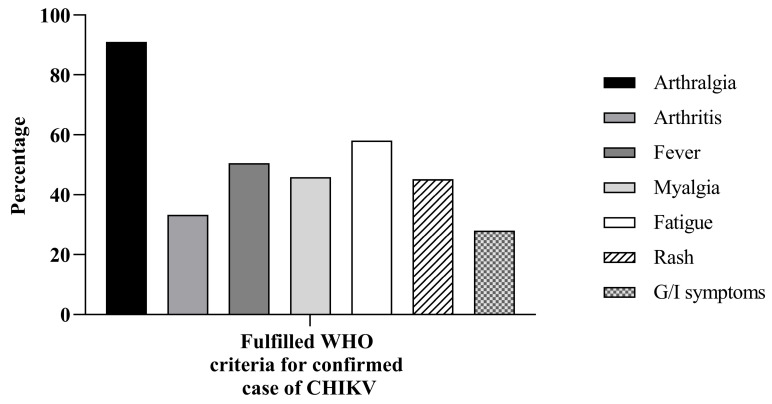
Clinical symptoms according to WHO criteria. WHO: World Health Organization, CHKV: chikungunya virus, G/I: gastrointestinal.

The most frequent symptom referred by patients was arthralgia (*n* = 254, 91.0%), followed by fatigue (*n* = 162, 58.1%), fever (*n* = 141, 50.5%), myalgia (*n* = 128, 45.9%), and rash (*n* = 126, 45.2%). The least frequent symptoms were arthritis and gastrointestinal, only present in 33.3% (*n* = 93) and 28.0% (*n* = 78) of the studied population, respectively. Figure 2 shows the clinical features in the studied patients.

### 3.3. Cytokine Measurements

Cytokine results were analyzed comparing patients who fulfilled the acute clinical case definition for CHIKV from the WHO, demographics (Supplementary Table S1), and clinical symptoms (Table 2).

High levels of FGF-2, GM-CSF, INF-α, IL-7, IL-8, IL-10, IL-12p40, MCP-1 and MIP1A were found in Caucasian patients with statistical significance. Conversely, Afro-American patients expressed statistically significant lower levels FGF-2, GM-CSF, INF-α, INF-γ, IL-1B, IL-6, IL-7, IL-8, IL-10, IL-12p40, IL-15, IL-17A, MCP-1, MIP-1A, MIP-1B and TNF-α. Patients with indigenous ethnicity expressed high levels of IL-8 and IP-10 with statistical significance, *p* < 0.05.

Low levels of IL-4 and IL-6 with statistical significance were found in patients with a high school education. Also, low levels of IL-15 were found in patients with a university education, with statistical significance, while statistically significant high levels of IL-4 and IL-10 were found in the same group. Patients with a bachelor’s degree had statistically significantly high levels of eotaxin.

Patients with low socioeconomic levels expressed low levels of IL-10 (in strata 1) and IL-4 (in strata 2) with statistical significance. High levels of eotaxin, FGF-2, INF-γ, IL-1RA, IL-6, IL-10, IL12p40, IL-12p70, IL-15, IL-17A, MIP-1B and TNF-α with statistical significance were found in patients living in the highest socioeconomic strata (strata 5). Patients with a special regime of health care expressed low levels of MIP-1A and MIP-1B with statistical significance.

Patients who fulfilled the WHO criteria for acute clinical case expressed high levels of GM-CSF, IL-1B, IL-4, IL-6, IL-15, IL-17A, MCP-1 and MIP-1A with statistical significance. Statistically significant low levels of FGF-2 and IL-7 were found in patients with arthritis. Patients with fever expressed statistically significantly high levels of IL-1B, IL-4, IL-6, IL-15, IL-17A, MCP-1 and MIP-1A and low levels of GM-CSF. High levels of INF-γ and IL-4 were found in patients with myalgia with statistical significance. Statistically significant low levels of IL-6 were found in the same patients with myalgia. In patients with fatigue, high levels of GM-CSF, INF-α, INF-γ, IL-1B, IL-4, IL-6, IL-7, IL-10, IL-12p40, IL-12p70, IL-15, IL-17A, MCP-1, MIP-1A, MIP-1B and TNF-α were found with statistical significance. Only IL-1RA was statistically significantly lower in these patients. Statistically significant high levels of GM-CSF, INF-γ, IL-4, IL-6, IL-12p70, IL-15, IL-17A, MCP-1, MIP-1A and MIP-1B were found in patients with rash. Patients with G/I symptoms expressed statistically significantly high levels of GM-CSF, INF-α, IL-1B, IL-2, IL-6, IL-7, IL-8, IL-10, IL-12p40, IL-15, IL-17A, MCP-1, MIP-1A, MIP-1B and TNF-α. In the same patients, statistically significant low levels of IL-4 and IL-12p70 were found.

**Figure 3:**
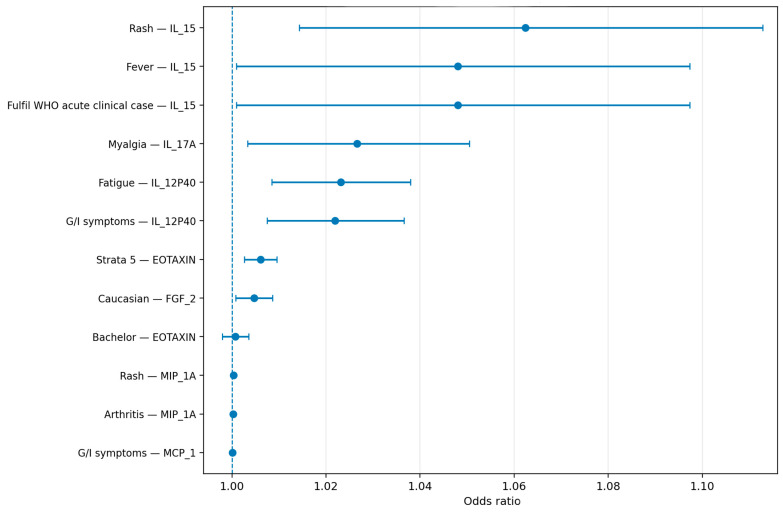
Forest plot of logistic regression analysis of cytokines and clinical/sociodemographic characteristics in patients with confirmed CHIKV infection.

To evaluate the association between cytokine concentrations and the main clinical and sociodemographic outcomes, logistic regression models were fitted using each outcome as a binary dependent variable. Cytokines were analyzed as continuous predictors, and odds ratios (ORs), 95% confidence intervals (CIs), and *p* values were estimated for each association. Multivariable backward stepwise logistic regression was used for outcomes in which more than one candidate predictor was considered, and the retained variables were reported as the final independent associations. For outcomes in which only one cytokine predictor was modeled or retained, the corresponding single-predictor logistic regression estimate was reported. Because cytokines were analyzed in their original continuous scale, each OR represents the change in the odds of the outcome for a one-unit increase in cytokine concentration. Accordingly, ORs were expected to be numerically close to 1.00, and were therefore reported with sufficient decimal precision to preserve interpretability. (Table 3, Figure 3).

Logistic regression analysis identified several cytokines associated with the principal clinical and sociodemographic outcomes in patients with confirmed CHIKV infection. Fulfillment of the WHO acute clinical case criteria was positively associated with IL-15, with an OR of 1.048 (95% CI 1.001–1.097, *p* = 0.045). Caucasian ethnicity was positively associated with FGF-2, with an OR of 1.005 (95% CI 1.001–1.009, *p* = 0.018). The highest socioeconomic stratum (strata 5) was positively associated with eotaxin, with an OR of 1.006 (95% CI 1.003–1.010, *p* < 0.001). Fever was also positively associated with IL-15, with an OR of 1.048 (95% CI 1.001–1.097, *p* = 0.045). Myalgia was positively associated with IL-17A, with an OR of 1.027 (95% CI 1.003–1.051, *p* = 0.025). Fatigue was positively associated with IL-12p40, with an OR of 1.023 (95% CI 1.009–1.038, *p* = 0.002). Rash was associated with both IL-15 and MIP-1A, with ORs of 1.062 (95% CI 1.014–1.113, *p* = 0.010) and 1.00036 (95% CI 1.00004–1.00068, *p* = 0.028), respectively. Gastrointestinal symptoms were associated with MCP-1 and IL-12p40, with ORs of 1.00014 (95% CI 1.00006–1.00023, *p* = 0.001) and 1.022 (95% CI 1.007–1.037, *p* = 0.003), respectively. (Table 3, Figure 3).

Not all evaluated associations reached statistical significance. Bachelor’s degree was not significantly associated with eotaxin (OR 1.001, 95% CI 0.998–1.004, *p* = 0.603), and arthritis was not significantly associated with MIP-1A (OR 1.00023, 95% CI 1.00000–1.00047, *p* = 0.054). These findings indicate that the most robust signals in the regression analysis were concentrated in IL-15, IL-17A, IL-12p40, MIP-1A, MCP-1, FGF-2, and eotaxin, particularly in relation to acute clinical expression, symptom burden, and selected sociodemographic characteristics. (Table 3, Figure 3).

Overall, the regression models supported a pattern in which higher levels of selected proinflammatory cytokines and chemokines were associated with greater odds of acute symptomatic CHIKV infection and with specific symptom domains, especially fever, myalgia, fatigue, rash, and gastrointestinal manifestations. Because these biomarkers were modeled as continuous variables, the effect sizes per unit increase were modest in numerical magnitude; however, the direction and statistical significance of the associations indicate biologically meaningful relationships between inflammatory activity and the clinical phenotype of infection (Table 3, Figure 3).

The main takeaways from the logistic regression analysis were that IL-15 emerged as the most consistent correlate of acute symptomatic disease, being associated with fulfillment of WHO acute clinical case criteria, fever, and rash. IL-17A was associated with myalgia, IL-12p40 with fatigue and gastrointestinal symptoms, MCP-1 with gastrointestinal symptoms, MIP-1A with rash, FGF-2 with Caucasian ethnicity, and eotaxin with the highest socioeconomic stratum. In contrast, the associations of eotaxin with bachelor’s degree and MIP-1A with arthritis were not statistically significant. Taken together, these findings indicate that a limited set of cytokines captures relevant biomolecular variation linked to the acute clinical presentation of CHIKV infection, while also suggesting that selected sociodemographic characteristics are associated with distinct immune-response profiles (Table 3, Figure 3).

### 3.4. Point-Biserial Analysis

Point-biserial analysis was made to analyze the correlations between cytokines, clinical symptoms, and demographic variables. In the analysis between cytokines (Figure 3 and Supplementary Table S2), IL-12p40 correlated strongly with 9 cytokines [FGF-2, INF-α, IL-7, IL-8, IL-10, IL-15, IL-17A, IP-10 and MCP-1]. Pearson values ranged from 0.42 to 0.85, all with *p* < 0.0001.

Strong correlations were found between IL-8 [GM-CSF, IL-6, IL-10, IL-12p40, IL-15, MCP-1, MIP-1A and TNF-α] and IL-15 [FGF-2, INF-α, IL-7, IL-8, IL-12p40, IL-17A, IP-10 and MCP-1] with 8 cytokines each. Pearson values ranged from 0.43 to 0.85, all with *p* < 0.0001.

Also, IL-12p40 [FGF-2, INF-α, IL-7, IL-8, IL-15, IL-17A, IP-10, MCP-1], and IL-15 [FGF-2, INF-α, IL-7, IL-8, IL-12p40, IL-17A, IP-10, MCP-1] share the same eight cytokines correlations except for IL-10 with IL-15 which the correlation is moderate (Pearson = 0.35, *p* < 0.0001) instead of strong like with IL-12p40 and IL-10 (Pearson = 0.461 *p* < 0.0001). GM-CSF and IL-6 showed strong correlations with seven similar cytokines, including each other (IL-1B, IL-8, IL-10, MIP-1A and MIP-1B). Pearson values ranged from 0.35 to 0.85, all with *p* < 0.000.

Five cytokines showed a strong correlation with six other cytokines. INF-α with FGF-2, IL-17, IL-12p40, IL-15, IL-17 and MCP-1; IL-10 with GM-CSF, IL-6, IL-8, IL-12p40, MCP-1, and MIP-1A; IL-17A with FGF-2, INF-α, INF-γ, IL-7, IL-12p40 and IL-15; MIP-1A with GM-CSF, IL-6, IL-8, IL-10, MIP-1B and TNF-α; and TNF-α with GM-CSF, IL-1B, IL-6, IL-8, MIP-1A and MIP-1B. Pearson values ranged from 0.47 to 0.85, all with *p* < 0.000.

FGF-2 and IL-7 showed a strong correlation with the same four cytokines (INF-α, IL-12p40, IL-15 and IL-17A). MIP-1B also showed a strong correlation with four cytokines [GM-CSF, IL-6, MIP-1A and TNF-α]. IL-1B was strongly correlated with GM-CSF, IL-6 and TNF-α, while IP-10 was correlated with IL-12p40 and IL-15. INF-γ was strongly correlated only with IL-17A. Eotaxin, IL-1RA, IL-2 and IL-4 had no strong correlations but moderate or weak correlations with other cytokines. Pearson values ranged from 0.42 to 0.73, all with *p* < 0.0001.

**Figure 4:**
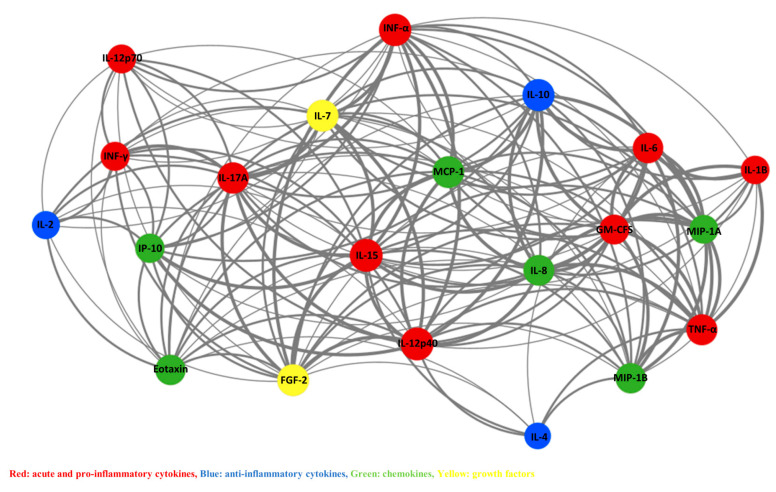
Cytokine correlations in patients with confirmed CHIKV infection. The strength of the connection is directly proportional to the thickness of the line, based on Pearson’s correlation coefficient.

There was no strong correlation when analyzing cytokine levels, clinical symptoms, and demographic variables (Table 4 and Supplementary Table S3). However, moderate correlations were found between MCP-1 and G/I symptoms, university education and IL-1B, eotaxin, and strata 5 and private health care, and strata 5 with eotaxin, IL-1B, IL-6, and TNF-α. Pearson values ranged from 0.21 to 0.36, all with *p* < 0.0001. Figure 4.

### 3.5. Cramer’s V Association

Cramér’s V coefficient was used to assess the strength of the association between categorical variables, providing a measure of effect size independent of sample size, with values ranging from 0 (no association) to 1 (perfect association). Strong correlations were found between arthritis and fulfilling WHO diagnostic criteria for CHIKV (OR: 8.3, CI: 4.5–15.2), fever (OR: 8.3, CI: 4.5–15.2) and fatigue (OR: 8.7, CI: 4.4–17.1). Also, strong correlations were found between fever and fulfilling WHO diagnostic criteria for CHIKV, arthritis (OR: 8.3, CI: 4.5–15.2), myalgia (OR: 8.2, CI: 4.7–14.1), fatigue (OR: 30.1, CI: 15.1–60.1), and rash (OR: 13.5, CI: 7.5–24.2). Myalgia showed strong correlations with fulfilling WHO diagnostic criteria for CHIKV (OR: 8.2, CI: 4.7–14.1), fever (OR: 8.2, CI: 4.7–14.1), fatigue (OR: 15.6, CI: 8.2–29.6), and rash (OR: 6.1, CI: 3.8–10.2). Fatigue was found to have strong correlations with all the clinical variables analyzed. Strong correlations were found between rash and fulfilling WHO diagnostic criteria for CHIKV (OR: 13.5, CI: 7.5–24.2), fever (OR: 13.5, CI: 7.5–24.2), myalgia (OR: 6.1, CI: 3.8–10.2), and fatigue (OR: 18.4, CI: 9.4–35.9). Finally, G/I symptoms showed a strong correlation only with fatigue (OR: 32.7, CI: 9.9–107). See Table 5 for details. Cramer’s V values ranged from 0.41 to 1.0, all with *p* < 0.0001.

When demographical variables were analyzed, a moderate correlation was found between strata 1 and subsidized health care (OR: 2.8, CI: 1.6–4.7), between strata 5 and Caucasians (OR: 0.36, CI: 0.3–0.4) and strata 5 and private health care (OR: 0.02, CI: 0.0–0.0) (Supplementary Table S4). Cramer’s V values ranged from 0.20 to 0.37, all with *p* ≤ 0.001.

The analysis of demographic and clinical symptom correlations (Supplementary Table S5) revealed that the lowest socioeconomical strata (strata 1) exhibited significant correlations with every symptom. Specifically, moderate correlations were observed between strata 1 and arthritis (OR: 2.6, CI: 1.5–4.3), fatigue (OR: 3.0, CI: 1.7–5.1), rash (OR: 2.6, CI: 1.6–4.3), and gastrointestinal symptoms (OR: 2.6, CI: 1.5–4.4). Also, having subsidized health care was correlated with fever (OR: 1.6, CI: 1.0–2.6), myalgia (OR: 1.6, CI: 1.0–2.7), fatigue (OR: 2.3, CI: 1.4–3.8), rash (OR: 1.8, CI: 1.1–2.9) and G/I symptoms (OR: 1.9, CI: 1.1–3.3). Interestingly, having a university education was correlated with arthritis (OR: 2.6, CI: 1.0–6.6), fever (OR: 9.9, CI: 2.2–43.7) and fatigue (OR: 3.0, CI: 1.0–9.5). Cramer’s V values ranged from 0.11 to 0.25.

### 3.6. Principal Component Analysis

**Figure 5:**
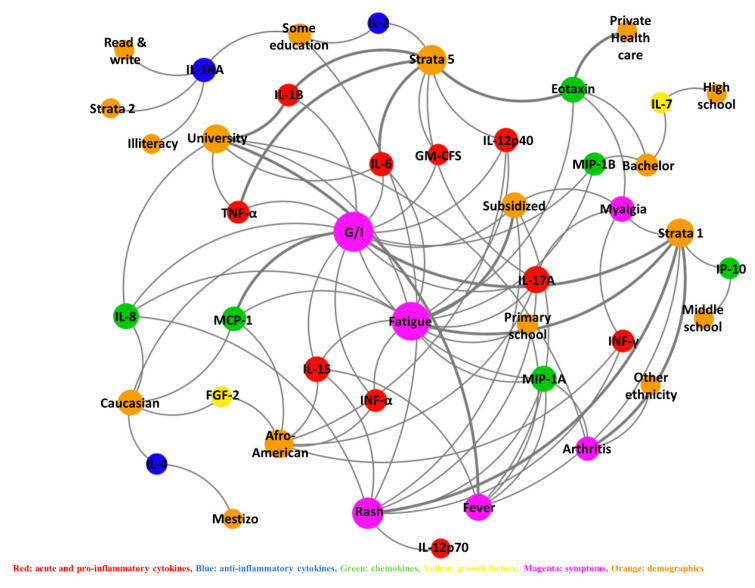
Correlations between cytokines, symptoms and demographics with confirmed CHIKV infection. The strength of the connection is directly proportional to the thickness of the line, based on Pearson’s correlation coefficient.

Principal component analysis identified three clusters at the optimal hierarchical level in the cytokine-only model. Cluster 1 comprised MIP-1A, GM-CSF, IL-6, and TNF-alpha; Cluster 2 comprised IL-15, IL-12, IFN-alpha, and FGF-2; and Cluster 3 comprised IL-10, IL-8, and MCP-1. Overall, Clusters 1 and 2 were composed predominantly of proinflammatory cytokines, whereas Cluster 3 combined chemokine activity with the anti-inflammatory cytokine IL-10, suggesting a distinct regulatory/chemotactic profile (Figure 5).

**Figure 6:**
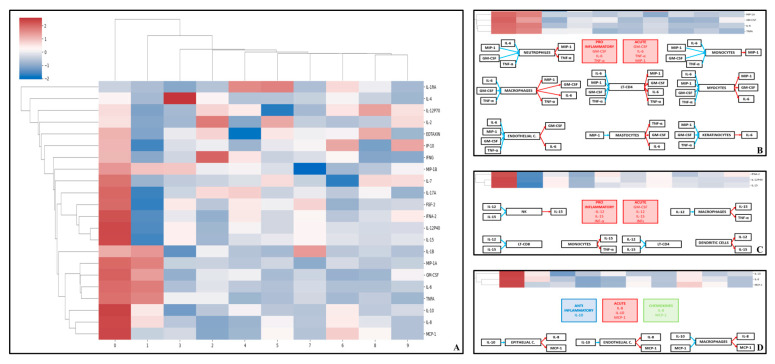
Clustering map of cytokine measurements in patients with confirmed CHIKV infection. (**A**) Clustering mal of cytokine measurements from PCA using 10 principal components. The clusters with the best hierarchical structure are shown in panels (**B**–**D**). A theoretical explanation for cytokine interaction on a cellular level of each cluster is depicted (**B**) Cluster of GM-CSF, MIP-1, IL-6 and TNF-α (**C**) Cluster of IL-12, IL-15, INF-α (**D**) Cluster of IL-8, IL-10 and MCP-1.

In the second phase, cytokine measurements and clinical variables were analyzed jointly. Three distinct cytokine-symptom patterns were identified. The cluster composed of IL-12, IL-15, and IFN-alpha was present in arthritis, fatigue, myalgia, rash, and gastrointestinal symptoms; however, in fever, all cytokines in this cluster were present except for IFN-α. A second cluster composed of GM-CSF, IL-6, and TNF-alpha was present in arthritis, fever, fatigue, myalgia, rash, and gastrointestinal symptoms. Finally, a third cluster composed of Eotaxin, IP-10, and IL-2 was present in arthritis and fatigue, whereas partial combinations of this cluster were observed in other symptoms. Specifically, Eotaxin and IP-10 were present in fever, IL-2 and IP-10 in myalgia, and Eotaxin and IL-2 in rash. Overall, arthritis and fatigue were the clinical manifestations that most consistently shared cytokine patterns across the three clusters (Figure 6).

**Figure 7:**
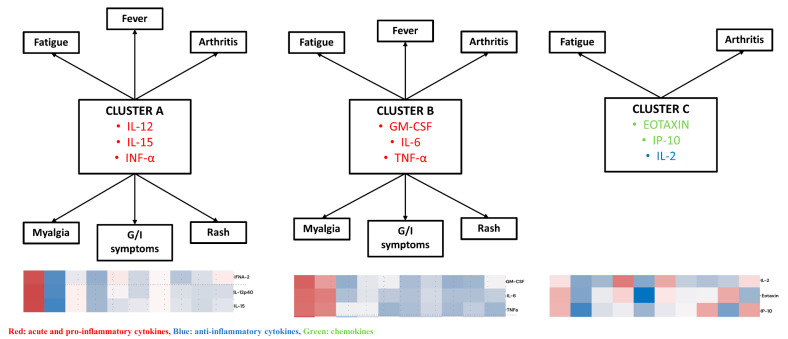
Clusters of cytokines and symptoms in patients with confirmed CHIKV infection.

**Figure 8:**
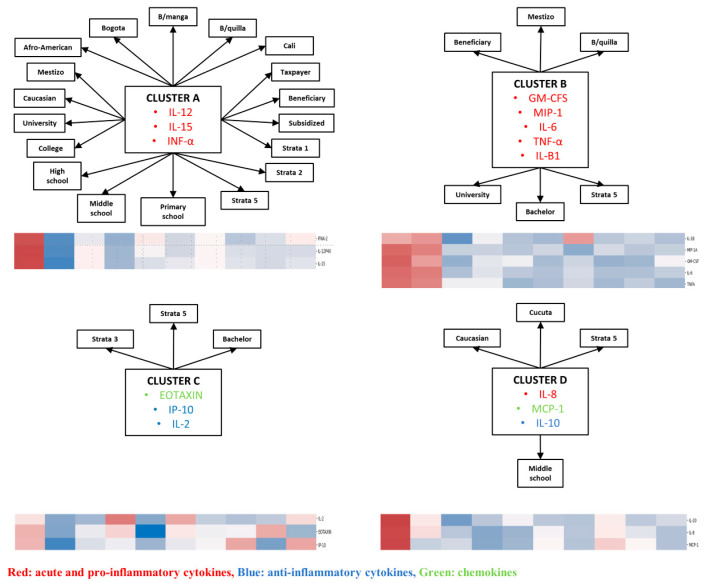
Clusters of cytokines and demographics in patients with confirmed CHIKV infection. Clusters were identified by integrating cytokine profiles and clinical features. Cytokines are color-coded as follows: red, pro-inflammatory; blue, anti-inflammatory; green, chemokines. Heatmaps show relative cytokine levels (red, higher; blue, lower; white, intermediate).

In the third phase, when cytokines were analyzed together with sociodemographic variables, four clusters were identified. In this phase, the manuscript uses a distinct lettering scheme (Clusters A–D). Cluster A comprised GM-CSF, TNF-alpha, IL-6, and MIP-1A; Cluster B comprised IL-12, IL-15, IFN-alpha, and FGF-2; Cluster C comprised IL-8, IL-10, and MCP-1; and Cluster D comprised Eotaxin, IL-2, and IP-10. Clusters A and B represented predominantly proinflammatory patterns, Cluster C represented an anti-inflammatory/chemokine profile, and Cluster D represented a mixed chemotactic/adaptive-response pattern (Figure 7 and Figure 8).

**Figure 9:**
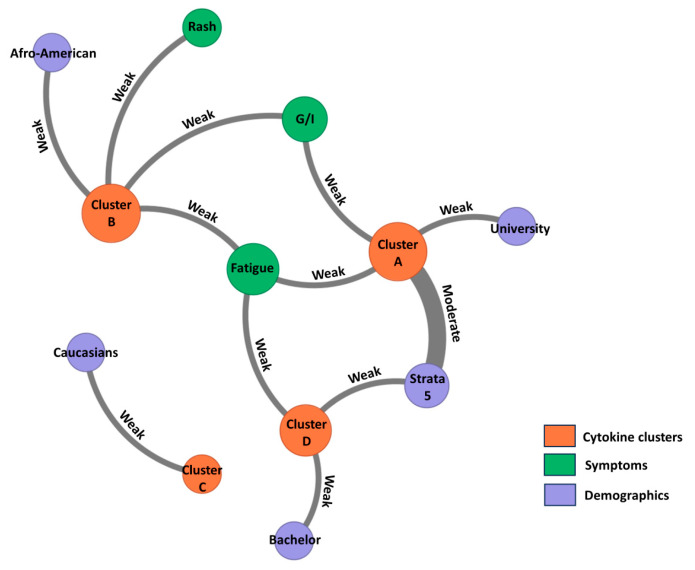
Clusters of cytokines and correlations with symptoms and demographics in patients with confirmed CHIKV infection. Cluster A: GM-CSF, TNF- α, IL-6, MIP-1A; Cluster B: IL-12, IL-15, INF- α, FGF-2; Cluster C: IL-8, IL-10, MCP-1; Cluster D: Eotaxin, IL-2, IP-10. Node connectors represent the strength of correlation between each node. The strength of the association is determined by Pearson’s correlation coefficient.

When correlations between these sociodemographic clusters and clinical or demographic variables were examined (Figure 9), a moderate correlation was found between Cluster A and socioeconomic stratum 5. In addition, Cluster A showed weak correlations with university education, fatigue, and gastrointestinal symptoms. Cluster B showed weak correlations with fatigue, gastrointestinal symptoms, rash, and Afro-American patients. Cluster D showed weak correlations with fatigue, socioeconomic strata 5, and having a bachelor’s degree, whereas Cluster C showed a weak correlation with Caucasian ethnicity only. Taken together, these findings suggest that CHIKV infection is characterized by recurrent but partially overlapping cytokine modules that persist across biological, clinical, and sociodemographic levels. Table 6.

## 4. Discussion

Framing these findings within the syndemic theory, this study illustrates how CHIKV infection does not occur in isolation [1,2,3,4,5]. The biological response (cytokine storm, specific pathway activation) interacts significantly with the clinical presentation (clustering of symptoms like fever, fatigue, myalgia, rash). Crucially, these bio-clinical interactions appear to be modulated by and potentially synergize with social and economic factors. The correlations observed between lower SES (strata 1, subsidized healthcare) and a higher prevalence of multiple symptoms (arthritis, fatigue, rash, G/I symptoms), alongside potential alterations in immune markers (lower IL-4/IL-10), suggest that social disadvantage may amplify the negative health impact of the infection. Similarly, the ethnic disparities observed in cytokine responses point to another layer of interaction where biological predispositions intersect with the infectious agent and potentially with unmeasured social or environmental factors associated with ethnicity in this context [18,19]. The strong correlations between clinical symptoms themselves (especially the centrality of fatigue) suggest potential reinforcing loops where one symptom may exacerbate another, further contributing to the overall disease burden experienced by individuals, particularly those facing social adversity.

Chikungunya virus (CHIKV) continues to pose a significant public health challenge globally, underscored by recent large outbreaks and the emergence of local transmission in previously unaffected regions like Argentina and Uruguay [20,21]. While the clinical manifestations and basic immune responses during acute CHIKV infection are increasingly characterized [6,7,8,22,23,24,25,26,27], the complex interplay between the virus, the host’s specific immune profile, and the broader socio-environmental context remains incompletely understood. The goal was to identify conditions exacerbating CHIKV’s impact in Colombia and understand the multidirectional causality inherent in syndemics to inform more effective and context-specific interventions.

Our cohort’s demographic profile—predominantly female, hispano, housewives from lower socioeconomic strata (SES) with subsidized healthcare, residing largely in Barranquilla—aligns with national Colombian patterns and previous reports pinpointing the Caribbean coast as heavily impacted during the epidemic [14,28,29]. The COPCORD sampling methodology likely contributed to the high representation of housewives. This demographic context is crucial within a syndemic framework, as factors like gender, occupation, income, healthcare access, and geographic location represent social and economic vulnerabilities that can interact with biological susceptibility to infection and disease severity [1,3,18]. The concentration of cases in specific regions like Barranquilla also points towards ecological factors (e.g., vector density, climate, urban infrastructure) that co-determine disease distribution alongside social and biological elements [29,30]. Understanding this baseline population context is essential for interpreting the observed biological and clinical variations.

A key finding was the significant difference in cytokine profiles based on ethnicity and SES. Caucasian patients exhibited higher levels of several proinflammatory cytokines (GM-CSF, IFN-α, IL-12p40), while African American patients showed broadly lower cytokine levels, both pro- and anti-inflammatory. These observations resonate with existing literature documenting ethnic variations in cytokine gene polymorphisms and baseline immune function, potentially influencing susceptibility to and severity of various diseases [31,32,33,34,35]. For instance, specific IFN-γ genotypes associated with lower expression are more prevalent in African Americans [33]. Our findings suggest these underlying genetic or epigenetic differences may modulate the immune response to CHIKV. Furthermore, the association of higher SES (strata 5) with elevated proinflammatory cytokines (FGF-2, IFN-γ, IL-1RA, IL-6, IL-12p40/p70, IL-15, IL-17A, MIP-1β, TNF-α) and lower SES (strata 1/2) with reduced anti-inflammatory cytokines (IL-10, IL-4) presents an intriguing contrast to some studies linking low SES primarily to heightened chronic inflammation (e.g., higher IL-6) [36]. However, the relationship between SES and acute inflammatory responses can be complex, potentially influenced by factors like nutrition, stress, co-morbidities, and even psychological resources, which might differ across SES levels [37,38].

These findings reinforce the interpretation that the acute clinical expression of CHIKV infection is closely linked to a restricted group of inflammatory and chemotactic mediators. IL-15 appeared as the most consistent marker, showing associations with WHO acute clinical case criteria, fever, and rash, which suggests a central role in the symptomatic inflammatory response. Likewise, the associations of IL-17A with myalgia, IL-12p40 with fatigue and gastrointestinal symptoms, and MCP-1 and MIP-1A with gastrointestinal symptoms and rash, respectively, support the view that specific cytokine pathways are related to distinct symptom clusters rather than to a nonspecific generalized response. The association of FGF-2 with Caucasian ethnicity and eotaxin with the highest socioeconomic stratum also suggests that host inflammatory patterns may vary according to sociodemographic context. Importantly, the use of greater numerical precision in reporting ORs and confidence intervals improves the interpretability of continuous biomarker models, since values close to 1.00 do not imply the absence of effect, but rather reflect the very small change in odds associated with a one-unit increase in cytokine concentration. Overall, these results strengthen the biological component of the proposed syndemic framework by showing that measurable immune mediators are linked not only to acute symptom expression, but also to selected social and demographic characteristics.

The clinical presentation in our cohort, dominated by arthralgia, fatigue, fever, myalgia, and rash, is consistent with classic acute CHIKV disease described globally [22,23,24,25,26,27]. Our analysis confirmed significant associations between these symptoms and elevated levels of acute-phase proinflammatory cytokines (GM-CSF, IFN-α, IL-1β, IL-6, IL-12p40/p70, IL-15, IL-17A, TNF-α) and chemokines (MCP-1, MIP-1α, MIP-1β, IL-8), alongside anti-inflammatory mediators (IL-4, IL-10) and growth factors (FGF-2, IL-7). This cytokine storm mirrors findings from other acute CHIKV cohorts, reflecting a robust innate and early adaptive immune activation crucial for viral control but also responsible for tissue damage and symptoms [19,39,40]. The multivariate regression pinpointed IL-15 (associated with fulfilling WHO criteria, fever, rash), IL-17A (myalgia), IL-12p40 (fatigue, G/I symptoms), MCP-1 (G/I symptoms), and MIP-1α (rash) as key correlates of specific clinical features. IL-15 is known to activate NK cells and T cells, IL-17A is involved in neutrophil recruitment and inflammation, IL-12 drives Th1 responses, while MCP-1 and MIP-1α are potent chemoattractants for monocytes/macrophages, highlighting the central role of these specific pathways in driving the symptomatic phase of CHIKV [19,41].

The network and principal component analyses provided deeper insights into the interconnectedness of the immune response and its links to clinical and demographic factors, aligning with a syndemic approach that emphasizes interactions. The strong correlations observed between multiple proinflammatory cytokines (e.g., IL-12p40, IL-15, IL-8, IL-6, GM-CSF, TNF-α hubs) suggest coordinated activation pathways, likely driven by macrophage activation, a central event in CHIKV pathogenesis [42,43]. The PCA identified distinct cytokine clusters: Cluster A (IL-12, IL-15, IFN-α) and B (GM-CSF, MIP-1, IL-6, TNF-α, IL-1B) represent core proinflammatory axes frequently associated with multiple symptoms (arthritis, fatigue, myalgia, rash, G/I symptoms) and demographic groups. Cluster C (Eotaxin, IP-10, IL-2) and D (IL-8, MCP-1, IL-10) might represent different facets or stages of the response, possibly involving endothelial activation, chemotaxis, and initial regulatory attempts [19,43]. The finding that fatigue and arthritis shared cytokine patterns across multiple clusters suggests common underlying inflammatory mechanisms for these debilitating symptoms. The weak but significant correlations linking these clusters to demographic variables (e.g., Cluster A with Strata 5; Cluster B with African American patients; Cluster D with bachelor’s degree) further reinforce the concept of interacting biological and social factors.

This multilevel analysis, demonstrating interconnectedness across biological (cytokines), clinical (symptoms), and sociodemographic (ethnicity, SES, education, healthcare) domains, supports the utility of a syndemic framework for understanding CHIKV in Colombia. It moves beyond a purely biomedical view to acknowledge that the severity and manifestation of infection are shaped by a complex interplay of factors operating at different levels [3,5]. Recognizing these interactions is vital for public health. Interventions solely focused on vector control or individual treatment may be insufficient if underlying social and economic drivers that increase vulnerability and potentially modulate immune responses are not addressed [1,18,44]. For instance, strategies might need to consider targeted support for low-SES communities, address healthcare access barriers, and investigate factors contributing to ethnic disparities in outcomes. Future research should longitudinally follow diverse cohorts to better understand the progression to chronic disease and how these syndemic interactions evolve over time, potentially incorporating additional environmental and psychosocial variables.

### Limitations

This study has limitations, including its cross-sectional design, which prevents causal inferences about the directionality of observed associations. The sample size for certain subgroups (e.g., highest SES, specific ethnicities) was small, limiting the generalizability of some findings related to these groups. While a comprehensive cytokine panel was used, other immune mediators and cell populations could also play important roles. Furthermore, detailed environmental data (e.g., housing quality, vector exposure) and psychosocial factors (e.g., stress, social support) were not included but recognized components of syndemic interactions [1,4]. Despite these limitations, this study provides valuable insights into the complex, multilevel interactions shaping CHIKV infection within a specific Colombian context, strongly advocating for a syndemic approach in future research and public health planning for this and similar infectious diseases.

The small sample size in the highest SES group requires caution in interpretation, but the data hint at complex interactions between social standing and acute immune reactivity during CHIKV infection, warranting further investigation in larger, diverse cohorts.

In Colombia, the prevalence of obesity is significantly higher in low socioeconomic status settings [45]. In recent years, some authors have described the role of obesity in arboviral diseases, noting that it not only acts as a confounding factor but also modulates the inflammatory immune response, thereby exacerbating viral pathogenesis and altering cytokine dynamics [46]. Due to the retrospective nature of the data collection, BMI was not available for this analysis, which constitutes a limitation of the present study. However, this highlights the need for future prospective studies on CHIKV fever that include immunological analyses incorporating cytokines and the metabolic and nutritional status of patients as critical variables for understanding the heterogeneity of the immune response in endemic populations in light of various socioeconomic factors.

## 5. Conclusions

In conclusion, this study highlights the complex syndemic nature of CHIKV infection in a Colombian cohort, demonstrating significant interplay between host immune responses, clinical manifestations, and sociodemographic factors. Key proinflammatory cytokine pathways are associated with acute symptoms, while notable differences in immune profiles linked to ethnicity and socioeconomic status suggest that these factors modulate the host response. Lower socioeconomic status (SES) was associated with a greater burden of symptoms, indicating that social disadvantage may exacerbate the clinical impact of CHIKV. These findings underscore the necessity of integrating social and biological perspectives to understand—and effectively address—CHIKV.

As global population mobility increases, arboviral infections such as CHIKV are more likely to spread beyond historically endemic regions, creating new diagnostic and management challenges for rheumatology services worldwide. Understanding how immune signatures interact with symptom phenotypes and social determinants is therefore essential not only for acute outbreak response, but also for anticipating and reducing longer-term rheumatologic burden.

Addressing social determinants of health—alongside traditional biomedical and vector control strategies—will be crucial for developing effective public health interventions to mitigate the impact of CHIKV and similar arboviral diseases, particularly among vulnerable populations. Further research employing longitudinal designs and incorporating a wider range of socio-environmental variables is warranted to fully elucidate these complex interactions and to inform integrated clinical and public health approaches in an increasingly interconnected world.

## Figures and Tables

**Table 1:** Demographics in patients with confirmed CHIKV infection.

	Total (*N* = 279)
Age in years (mean ± SD)	48.1 ± 17.7
Gender	
Female	195 (69.9%)
Ethnicity	
Hispano	133 (47.7%)
Caucasian	106 (38.0%)
Afro-American	29 (10.4%)
Indigenous	8 (2.9%)
Other	3 (1.1%)
City	
Barranquilla	155 (55.6%)
Bogotá	44 (15.8%)
Cúcuta	32 (11.5%)
Medellín	17 (6.1%)
Cali	17 (6.1%)
Bucaramanga	14 (5.0%)
Educational level	
None	20 (7.2%)
Some type of education	259 (92.8%)
Working status	
None	17 (6.1%)
Some type of work	262 (93.9%)
Health care	
Public health care	
Taxpayer	53 (19.0%)
Beneficiary	61 (21.9%)
Subsidized	162 (58.1%)
Special regime	2 (0.7%)
Private health care	1 (0.4%)
Social strata	
Strata 1	107 (38.4%)
Strata 2	99 (35.5%)
Strata 3	57 (20.4%)
Strata 4	9 (3.2%)
Strata 5	7 (2.5%)

CHIKV: chikungunya virus; World Health Organization Criteria for confirmed case of CHIKV; SD: standard deviation; USD: United States dollars.

**Table 2:** Mean cytokine levels in patients with confirmed CHIKV infection.

	Fulfill WHO Acute Clinical Case	Arthralgia (*n* = 254)	Arthritis (*n* = 93)	Fever (*n* = 141)	Myalgia (*n* = 128)	Fatigue (*n* = 162)	Rash (*n* = 126)	G/I Symptoms (*n* = 78)	Total (*n* = 279)
Eotaxin	201.4	188.8	204.1	190.9	204.0	201.6	199.1	191.2	184.8
FGF-2	90.9	88.4	76.5 *	89.5	90.1	90.1	89.2	89.4	87.7
GM-CSF	24.4 *	20.2	18.0	18.3 *	18.7	25.6 *	20.5 *	37.8 *	19.3
INF-α	48.8	44.4	37.7	47.6	47.4	49.2 *	48.8	51.8 *	43.6
INF-γ	16.9	15.9	14.3	16.2	18.0 *	16.4 *	18.3 *	17.2	15.5
IL-1B	159.4 *	115.3	238.2	164.5 *	169.7	175.8 *	184.9	333.0 *	106.0
IL-1RA	309.4	372.5	458.1	337.5	355.4	334.4 *	366.9	185.4	346.0
IL-2	3.5	3.3	3.4	3.4	3.5	3.4	3.7	3.5 *	3.4
IL-4	249.9 *	246.0	224.2	277.0 *	202.4 *	250.9 *	264.4 *	229.8 *	235.0
IL-6	167.6 *	127.1	182.6	159.8 *	96.0 *	182.8 *	182.8 *	247.8 *	117.0
IL-7	17.8	15.8	13.8 *	17.5	17.0	17.4 *	18.2	19.6 *	16.3
IL-8	4126.9	3429.0	3779.6	3773.5	3269.4	4423.4	4596.3	5615.1 *	3269.8
IL-10	19.8	19.0	18.2	18.5	17.6	20.7 *	18.0	23.6 *	18.3
IL-12p40	21.2	19.1	16.3	19.9	20.2	21.6 *	20.8	24.0 *	18.7
IL-12p70	13.5	12.2	12.4	13.5	13.2	13.5 *	14.5 *	11.3 *	11.9
IL-15	8.3 *	7.5	6.7	8.1 *	8.0	8.2 *	8.4 *	8.8 *	7.4
IL-17A	14.6 *	13.4	11.5	13.8 *	14.8	14.3 *	14.9 *	15.5 *	13.1
IP-10	683.2	667.6	596.6	673.2	712.0	683.1	720.5	553.3	655.5
MCP-1	2574.3 *	2254.4	2177.5	2524.8 *	2235.5	2653.5 *	2468.3	3202.5 *	2206.3
MIP-1A	406.2 *	303.7	474.4	414.3 *	323.1	435.6 *	470.0 *	550.1 *	289.5
MIP-1B	569.8	441.9	480.3	558.8	234.6	608.6 *	589.8 *	940.7 *	417.5
TNF-α	95.9	76.5	119.7	101.5	65.0	102.0 *	112.2	146.6 *	72.2

Values in pg/L; CHIKV: chikungunya virus; WHO: World Health Organization. * Student’s *t*-test *p* < 0.05. Comparison of cytokine levels and symptoms as defined by WHO criteria. The value *n* corresponds to the total number of patients who exhibited the symptom.

**Table 3:** Logistic regression analysis of cytokines associated with clinical and sociodemographic characteristics in patients with confirmed CHIKV infection.

Outcome	Predictor	OR	95% CI	*p* Value	Source
Fulfill WHO acute clinical case	IL-15	1.04804	1.00096–1.09735	0.0454	Backward multivariable model
Caucasian	FGF-2	1.00472	1.00082–1.00863	0.0176	Backward multivariable model
Bachelor	Eotaxin	1.00074	0.99795–1.00355	0.6026	Single-predictor logistic model
Strata 5	Eotaxin	1.00608	1.00265–1.00952	0.0005	Single-predictor logistic model
Arthritis	MIP-1A	1.00023	1.00000–1.00047	0.0537	Single-predictor logistic model
Fever	IL-15	1.04804	1.00096–1.09735	0.0454	Backward multivariable model
Myalgia	IL-17A	1.02664	1.00331–1.05051	0.0250	Single-predictor logistic model
Fatigue	IL-12p40	1.02316	1.00852–1.03801	0.0018	Backward multivariable model
Rash	IL-15	1.06241	1.01429–1.11281	0.0105	Single-predictor logistic model
Rash	MIP-1A	1.00036	1.00004–1.00068	0.0278	Backward multivariable model
G/I symptoms	MCP-1	1.00014	1.00006–1.00023	0.0011	Backward multivariable model
G/I symptoms	IL-12p40	1.02194	1.00749–1.03659	0.0028	Single-predictor logistic model

Odds ratios (ORs), 95% confidence intervals (CIs), and *p* values are shown for the cytokine predictors included in the final logistic regression models. Cytokines were analyzed as continuous variables; therefore, each OR represents the change in the odds of the corresponding outcome for a one-unit increase in cytokine concentration. Because these biomarkers were measured on a fine continuous scale, several ORs are numerically close to 1.00 and should be interpreted together with their confidence intervals and *p*-values. CHIKV: chikungunya virus; WHO: World Health Organization; G/I: gastrointestinal.

**Table 4:** Point-biserial correlation analysis of clinical symptoms and cytokines.

	Fulfill WHO Acute Clinical Case	Arthritis (*n* = 254)	Fever (*n* = 141)	Myalgia (*n* = 128)	Fatigue (*n* = 162)	Rash (*n* = 126)	G/I Symptoms (*n* = 78)
Eotaxin	Pearson = 0.042 *p* = 0.483	Pearson = 0.093 *p* = 0.121	Pearson = 0.042 *p* = 0.483	Pearson = 0.120 *p* = 0.044	Pearson = 0.134 *p* = 0.025	Pearson = 0.088 *p* = 0.142	Pearson = 0.027 *p* = 0.655
FGF-2	Pearson = 0.026 *p* = 0.662	Pearson = 0.116 *p* = 0.053	Pearson = 0.026 *p* = 0.662	Pearson = 0.032 *p* = 0.589	Pearson = 0.041 *p* = 0.491	Pearson = 0.019 *p* = 0.749	Pearson = 0.016 *p* = 0.795
GM-CSF	Pearson = 0.015 *p* = 0.800	Pearson = 0.014 *p* = 0.819	Pearson = 0.004 *p* = 0.800	Pearson = 0.009 *p* = 0.878	Pearson = 0.110 *p* = 0.066	Pearson = 0.015 *p* = 0.797	Pearson = 0.172 *p* = 0.004
INF-α	Pearson = 0.096 *p* = 0.110	Pearson = 0.102 *p* = 0.090	Pearson = 0.096 *p* = 0.110	Pearson = 0.082 *p* = 0.173	Pearson = 0.155 *p* = 0.009	Pearson = 0.111 *p* = 0.064	Pearson = 0.122 *p* = 0.042
INF-γ	Pearson = 0.032 *p* = 0.600	Pearson = 0.049 *p* = 0.417	Pearson = 0.032 *p* = 0.600	Pearson = 0.123 *p* = 0.040	Pearson = 0.053 *p* = 0.382	Pearson = 0.134 *p* = 0.025	Pearson = 0.056 *p* = 0.354
IL-1B	Pearson = 0.052 *p* = 0.388	Pearson = 0.082 *p* = 0.171	Pearson = 0.052 *p* = 0.388	Pearson = 0.051 *p* = 0.392	Pearson = 0.072 *p* = 0.230	Pearson = 0.063 *p* = 0.296	Pearson = 0.124 *p* = 0.038
IL-1RA	Pearson = 0.003 *p* = 0.954	Pearson = 0.032 *p* = 0.595	Pearson = 0.003 *p* = 0.954	Pearson = 0.003 *p* = 0.954	Pearson = 0.006 *p* = 0.927	Pearson = 0.008 *p* = 0.899	Pearson = 0.040 *p* = 0.502
IL-2	Pearson = 0.002 *p* = 0.977	Pearson = 0.004 *p* = 0.950	Pearson = 0.002 *p* = 0.977	Pearson = 0.038 *p* = 0.532	Pearson = 0.009 *p* = 0.880	Pearson = 0.073 *p* = 0.255	Pearson = 0.011 *p* = 0.854
IL-4	Pearson = 0.069 *p* = 0.252	Pearson = 0.012 *p* = 0.836	Pearson = 0.069 *p* = 0.252	Pearson = 0.049 *p* = 0.417	Pearson = 0.030 *p* = 0.615	Pearson = 0.043 *p* = 0.473	Pearson = 0.005 *p* = 0.930
IL-6	Pearson = 0.070 *p* = 0.241	Pearson = 0.075 *p* = 0.209	Pearson = 0.070 *p* = 0.241	Pearson = 0.031 *p* = 0.601	Pearson = 0.126 *p* = 0.035	Pearson = 0.097 *p* = 0.105	Pearson = 0.133 *p* = 0.027
IL-7	Pearson = 0.067 *p* = 0.266	Pearson = 0.101 *p* = 0.092	Pearson = 0.067 *p* = 0.266	Pearson = 0.034 *p* = 0.572	Pearson = 0.071 *p* = 0.237	Pearson = 0.094 *p* = 0.118	Pearson = 0.114 *p* = 0.057
IL-8	Pearson = 0.063 *p* = 0.297	Pearson = 0.044 *p* = 0.461	Pearson = 0.063 *p* = 0.297	Pearson = 0.000 *p* = 0.999	Pearson = 0.167 *p* = 0.005	Pearson = 0.148 *p* = 0.013	Pearson = 0.180 *p* = 0.003
IL-10	Pearson = 0.004 *p* = 0.941	Pearson = 0.003 *p* = 0.957	Pearson = 0.004 *p* = 0.941	Pearson = 0.019 *p* = 0.757	Pearson = 0.080 *p* = 0.181	Pearson = 0.009 *p* = 0.875	Pearson = 0.093 *p* = 0.121
IL-12p40	Pearson = 0.065 *p* = 0.278	Pearson = 0.095 *p* = 0.113	Pearson = 0.065 *p* = 0.278	Pearson = 0.078 *p* = 0.191	Pearson = 0.190 *p* = 0.001	Pearson = 0.104 *p* = 0.082	Pearson = 0.183 *p* = 0.002
IL-12p70	Pearson = 0.084 *p* = 0.164	Pearson = 0.018 *p* = 0.771	Pearson = 0.084 *p* = 0.164	Pearson = 0.059 *p* = 0.329	Pearson = 0.095 *p* = 0.112	Pearson = 0.123 *p* = 0.040	Pearson = 0.02 *p* = 0.720
IL-15	Pearson = 0.121 *p* = 0.0.43	Pearson = 0.099 *p* = 0.101	Pearson = 0.121 *p* = 0.043	Pearson = 0.091 *p* = 0.129	Pearson = 0.177 *p* = 0.003	Pearson = 0.156 *p* = 0.009	Pearson = 0.158 *p* = 0.008
IL-17a	Pearson = 0.067 *p* = 0.265	Pearson = 0.106 *p* = 0.076	Pearson = 0.067 *p* = 0.265	Pearson = 0.138 *p* = 0.021	Pearson = 0.128 *p* = 0.032	Pearson = 0.144 *p* = 0.016	Pearson = 0.135 *p* = 0.024
IP-10	Pearson = 0.031 *p* = 0.607	Pearson = 0.072 *p* = 0.228	Pearson = 0.031 *p* = 0.607	Pearson = 0.090 *p* = 0.133	Pearson = 0.056 *p* = 0.349	Pearson = 0.102 *p* = 0.088	Pearson = 0.111 *p* = 0.065
MCP-1	Pearson = 0.109 *p* = 0.069	Pearson = 0.007 *p* = 0.908	Pearson = 0.109 *p* = 0.069	Pearson = 0.009 *p* = 0.880	Pearson = 0.178 *p* = 0.003	Pearson = 0.080 *p* = 0.180	Pearson = 0.210 *p* < 0.000
MIP-1A	Pearson = 0.120 *p* = 0.046	Pearson = 0.124 *p* = 0.038	Pearson = 0.120 *p* = 0.046	Pearson = 0.029 *p* = 0.626	Pearson = 0.163 *p* = 0.006	Pearson = 0.155 *p* = 0.009	Pearson = 0.154 *p* = 0.01
MIP-1B	Pearson = 0.076 *p* = 0.206	Pearson = 0.024 *p* = 0.695	Pearson = 0.076 *p* = 0.206	Pearson = 0.090 *p* = 0.136	Pearson = 0.119 *p* = 0.046	Pearson = 0.083 *p* = 0.166	Pearson = 0.173 *p* = 0.004
TNF-α	Pearson = 0.026 *p* = 0.662	Pearson = 0.106 *p* = 0.076	Pearson = 0.094 *p* = 0.119	Pearson = 0.021 *p* = 0.725	Pearson = 0.111 *p* = 0.064	Pearson = 0.115 *p* = 0.055	Pearson = 0.147 *p* = 0.014

CHIKV: Chikungunya virus; WHO: World Health Organization; G/I: gastrointestinal. The value *n* corresponds to the total number of patients who exhibited the symptom. Pearson correlation coefficient. Cells are color-coded according to statistical significance and strength of correlation. Red indicates statistically significant correlations (*p* < 0.05), with darker shades representing higher correlation coefficients. Orange indicates statistically significant correlations with lower magnitude. White indicates non-significant correlations (*p* ≥ 0.05).

**Table 5:** Cramer’s correlation analysis of clinical symptoms.

	Fulfill WHO Acute Clinical Case	Arthritis (*n* = 254)	Fever (*n* = 141)	Myalgia (*n* = 128)	Fatigue (*n* = 162)	Rash (*n* = 126)	G/I Symptoms (*n* = 78)
Fulfill WHO acute clinical case		Cramer’s = 0.44 *p* < 0.000 OR: 8.3 (4.5–15.2)	Cramer’s = 1.0 *p* < 0.000	Cramer’s = 0.47 *p* < 0.000 OR: 8.2 (4.7–14.1)	Cramer’s = 0.67 *p* < 0.000 OR: 30.2 (15.1–60.1)	Cramer’s = 0.56 *p* < 0.000 OR: 13.5 (7.5–24.2)	Cramer’s = 0.28 *p* < 0.000 OR: 3.7 (2.1–6.7)
Arthritis	Cramer’s = 0.44 *p* < 0.000 OR: 8.3 (4.5–15.2)		Cramer’s = 0.44 *p* < 0.000 OR: 8.32 (4.5–15.2)	Cramer’s = 0.34 *p* < 0.000 OR: 4.5 (2.6–7.7)	Cramer’s = 0.41 *p* < 0.000 OR: 8.7 (4.4–17.1)	Cramer’s = 0.26 *p* < 0.000 OR: 3.1 (1.8–5.1)	Cramer’s = 0.08 *p* = 0.15 OR: 1.4 (0.8–2.5)
Fever	Cramer’s = 1.0 *p* < 0.000	Cramer’s = 0.44 *p* < 0.000 OR: 8.3 (4.5–15.2)		Cramer’s = 0.47 *p* < 0.000 OR: 8.2 (4.7–14.1)	Cramer’s = 0.67 *p* < 0.000 OR: 30.1 (15.1–60.1)	Cramer’s = 0.56 *p* < 0.000 OR: 13.5 (7.5–24.2)	Cramer’s = 0.28 *p* < 0.000 OR: 3.7 (2.1–6.7)
Myalgia	Cramer’s = 0.47 *p* < 0.000 OR: 8.2 (4.7–14.1)	Cramer’s = 0.34 *p* < 0.000 OR: 4.5 (2.6–7.7)	Cramer’s = 0.47 *p* < 0.000 OR: 8.2 (4.7–14.1)		Cramer’s = 0.56 *p* < 0.000 OR: 15.6 (8.2–29.6)	Cramer’s = 0.42 *p* < 0.000 OR: 6.1 (3.8–10.2)	Cramer’s = 0.13 *p* = 0.028 OR: 1.8 (1.1–3.0)
Fatigue	Cramer’s = 0.67 *p* < 0.000 OR: 30.2 (15.1–60.1)	Cramer’s = 0.41 *p* < 0.000 OR: 8.7 (4.4–17.1)	Cramer’s = 0.67 *p* < 0.000 OR: 30.1 (15.1–60.1)	Cramer’s = 0.56 *p* < 0.000 OR: 15.6 (8.2–29.6)		Cramer’s = 0.58 *p* < 0.000 OR: 18.4 (9.4–35.9)	Cramer’s = 0.48 *p* < 0.000 OR: 32.7 (9.9–107)
Rash	Cramer’s = 0.56 *p* < 0.000 OR: 13.5 (7.5–24.2)	Cramer’s = 0.26 *p* < 0.000 OR: 3.1 (1.8–5.1)	Cramer’s = 0.56 *p* < 0.000 OR: 13.5 (7.5–24.2)	Cramer’s = 0.42 *p* < 0.000 OR: 6.1 (3.8–10.2)	Cramer’s = 0.58 *p* < 0.000 OR: 18.4 (9.4–35.9)		Cramer’s = 0.30 *p* < 0.000 OR: 4.0 (2.3–7.0)
G/I symptoms	Cramer’s = 0.28 *p* < 0.000 OR: 3.7 (2.1–6.7)	Cramer’s = 0.08 *p* = 0.15 OR: 1.4 (0.8–2.5)	Cramer’s = 0.28 *p* < 0.000 OR: 3.7 (2.1–6.7)	Cramer’s = 0.13 *p* = 0.028 OR: 1.8 (1.1–3.0)	Cramer’s = 0.48 *p* < 0.000 OR: 32.7 (9.9–107)	Cramer’s = 0.30 *p* < 0.000 OR: 4.0 (2.3–7.0)	

CHIKV: Chikungunya virus; WHO: World Health Organization; G/I: gastrointestinal; Green: strong correlation; Yellow: moderate correlation; Red: weak correlation. The value *n* corresponds to the total number of patients who exhibited the symptom.

**Table 6:** Characteristics of cytokine clusters identified in the biological and clinical phases of the principal component analysis in patients with confirmed CHIKV infection.

Cluster	Main Components	Analytical Phase	Main Associated Pattern	Interpretation
Cluster 1	MIP-1A, GM-CSF, IL-6, TNF-alpha	Phase I: cytokines only	Predominantly proinflammatory cytokine cluster	Represents a core inflammatory response module in the biological-only analysis
Cluster 2	IL-15, IL-12, IFN-alpha, FGF-2	Phase I: cytokines only	Predominantly proinflammatory cytokine cluster	Represents a second immune-activation module distinct from Cluster 1
Cluster 3	IL-10, IL-8, MCP-1	Phase I: cytokines only	Anti-inflammatory/chemokine cluster	Suggests a regulatory/chemotactic response pattern
Clinical cluster pattern 1	IL-12, IL-15, IFN-alpha	Phase II: cytokines + clinical variables	Present in arthritis, fatigue, myalgia, rash, and G/I symptoms; in fever, the pattern is retained except for IFN-alpha	Represents a recurrent proinflammatory symptom-linked cytokine pattern
Clinical cluster pattern 2	GM-CSF, IL-6, TNF-alpha	Phase II: cytokines + clinical variables	Present in arthritis, fever, fatigue, myalgia, rash, and G/I symptoms	Represents a broadly shared inflammatory pattern across acute clinical manifestations
Clinical cluster pattern 3	Eotaxin, IP-10, IL-2	Phase II: cytokines + clinical variables	Present in arthritis and fatigue; partial combinations observed in fever, myalgia, and rash	Represents a more selective chemotactic/adaptive-response pattern associated with specific symptoms

Abbreviations: CHIKV, chikungunya virus; IFN-alpha, interferon alpha; GM-CSF, granulocyte-macrophage colony-stimulating factor; MCP-1, monocyte chemoattractant protein-1; MIP-1A, macrophage inflammatory protein-1A; G/I, gastrointestinal. Phase I included cytokines only; Phase II integrated cytokines with clinical variables.

## Data Availability

The data that support the findings of this study are available from the corresponding author, J.L., upon reasonable request.

## Supplementary Materials

The following supporting information can be downloaded at: https://www.mdpi.com/article/10.3390/v18050549/s1. Figure S1: Map of the cities studied that received a positive COPCORD score. COPCORD: Community Program for the Control of Rheumatic Diseases, Table S1: Cytokine values and demographics in patients with confirmed CHIKV infection, Table S2: Point-biserial correlation analysis of cytokines in patients with confirmed CHIKV infection, Table S3: Point-biserial correlation analysis of demographics and cytokines in patients with confirmed CHIKV infection, Table S4: Cramer’s correlation analysis of demographics in patients with confirmed CHIKV infection, Table S5: Cramer’s correlation analysis of demographics and clinical symptoms in patients with confirmed CHIKV infection.

## Abbreviations

The following abbreviations are used in this manuscript:

SES	Socioeconomic status
CHIKV	Chikungunya virus
HIV	Human immunodeficiency virus
IgM	Immunoglobulin M
IgG	Immunoglobulin G
RNA	Ribonucleic acid
IL	Interleukin
TNF	Tumor necrosis factor
IFN	Interferon
MCP	Monocyte chemoattractant protein
GM-CSF	Granulocyte-macrophage colony-stimulating factor
VAS	Visual analog scale
SD	Standard deviation
CI	Confidence interval
OR	Odds ratio
RR	Relative risk
BMI	Body mass index
CRP	C-reactive protein
ESR	Erythrocyte sedimentation rate
WHO	World Health Organization
DENV	Dengue virus
ZIKV	Zika virus
FGF-2	Fibroblast growth factor 2
G/I	Gastrointestinal
IFN-α	Interferon alpha
IFN-γ	Interferon gamma
IL-1β	Interleukin 1 beta
IL-1RA	Interleukin 1 receptor antagonist
IL-12p40	Interleukin 12 subunit p40
IL-12p70	Interleukin 12 subunit p70
IL-17A	Interleukin 17A
IP-10	Interferon gamma-induced protein 10
MCP-1	Monocyte chemoattractant protein 1
MIP-1A	Macrophage inflammatory protein 1 alpha
MIP-1B	Macrophage inflammatory protein 1 beta
TNF-α	Tumor necrosis factor alpha
