# Supplementary material for: Intersecting Epidemics: A Multilevel Syndemic Analysis of a Chikungunya Virus Epidemic in Colombia Through Clinical, Biological, and Socioeconomic Factors
Source: Viruses. 2026 May 9;18(5):549. doi: 10.3390/v18050549 (PMC13211427; doi:10.3390/v18050549)
Supplement: Supplementary file 1 [file viruses-18-00549-s001.zip › viruses-4215431-supplementary.pdf]

**Supplementary Tables:**

**Supplementary Table S1.** Cytokine values and demographics in patients with confirmed CHIKV infection part 1

| | Ethnicity | | | | | Literacy | | Educational level | | | | | |
|---|---|---|---|---|---|---|---|---|---|---|---|---|---|
| | Mestizo | Caucasian | Afro-American | Indigenous | Other | Read & writes | Illiteracy | Some education | Primary school | Middle school | High school | Bachelor | University |
| Eotaxin | 179.8 | 191.7 | 163.0 | 283.0 | 111.5 | 186.7 | 155.4 | 187.2 | 183.6 | 180.4 | 157.7 | 247.7* | 201.3 |
| FGF-2 | 85.0 | 100.8* | 58.5* | 72.2 | 64.1 | 87.7 | 86.6 | 87.1 | 91.5 | 92.3 | 79.3 | 82.9 | 93.5 |
| GM-CFS | 22.0 | 19.9* | 8.4* | 9.7 | 10.2 | 19.4 | 17.2 | 19.6 | 16.3 | 14.7 | 11.9 | 36.9 | 36.7 |
| INF-$\alpha$ | 43.3 | 48.3* | 25.6* | 54.0 | 40.5 | 43.5 | 44.8 | 43.4 | 41.6 | 47.7 | 40.0 | 43.3 | 56.2 |
| INF-$\gamma$ | 17.7 | 15.2 | 7.6* | 15.8 | 7.5 | 15.7 | 13.0 | 15.5 | 14.0 | 17.5 | 16.9 | 13.5 | 16.8 |
| IL-1B | 60.7 | 200.1 | 5.1* | 16.2 | 3.4 | 111.8 | 16.1 | 113.0 | 35.2 | 21.5 | 11.9 | 122.0 | 979.7 |
| IL-1RA | 151.9 | 689.6 | 83.7 | 82.0 | 47.4 | 249.4 | 1834.3 | 249.6 | 127.1 | 138.0 | 519.5 | 157.8 | 209.0 |
| IL-2 | 3.3 | 3.7 | 2.9 | 2.3 | 2.6 | 3.4 | 3.1 | 3.3 | 3.1 | 3.2 | 3.2 | 4.1 | 2.7 |
| IL-4 | 312.4 | 137.6 | 283.6 | 85.8 | 172.8 | 240.5 | 149.8 | 239.7 | 270.3 | 235.8 | 178.6* | 316.1 | 203.8* |
| IL-6 | 155.2 | 108.2 | 13.9* | 8.6 | 16.8 | 121.6 | 45.5 | 122.0 | 83.2 | 60.4 | 18.4* | 260.5 | 509.3 |
| IL-7 | 15.9 | 18.6* | 10.8* | 12.8 | 13.0 | 16.6 | 11.8 | 16.6 | 14.7 | 18.1 | 12.6 | 23.4 | 22.3* |
| IL-8 | 2885.2 | 4788.4* | 497.3* | 814.2* | 12.6 | 3390.9 | 1403.4 | 3418.7 | 3600.6 | 4922.1 | 1749.6 | 2950.6 | 6855.3 |
| IL-10 | 20.1 | 20.1* | 7.3* | 9.2 | 7.6 | 18.4 | 17.5 | 18.4 | 17.8 | 21.0 | 12.8 | 21.9 | 30.4* |
| IL-12p40 | 18.7 | 21.3* | 10.9* | 11.4 | 18.6 | 18.6 | 19.9 | 18.9 | 19.6 | 20.9 | 15.9 | 18.8 | 23.5 |
| IL-12p70 | 11.5 | 12.2 | 12.8 | 14.8 | 6.9 | 12.1 | 10.1 | 11.8 | 12.7 | 11.5 | 9.6 | 14.8 | 10.4 |
| IL-15 | 7.6 | 8.0 | 5.0* | 6.1 | 6.4 | 7.53 | 6.1 | 7.5 | 7.3 | 8.7 | 6.6 | 7.8 | 9.5* |

| | | | | | | | | | | | | | |
|---|---|---|---|---|---|---|---|---|---|---|---|---|---|
| IL-17A | 13.7 | 13.6 | 8.7* | 13.0 | 11.0 | 13.1 | 13.1 | 13.1 | 13.4 | 13.6 | 12.2 | 13.0 | 14.6 |
| IP-10 | 700.0 | 654.2 | 487.6 | 701.6* | 230.6 | 659.6 | 592.9 | 662.0 | 663.9 | 827.4 | 557.4 | 725.6 | 578.1 |
| MCP-1 | 2101.9 | 2793.0* | 965.2* | 1191.5 | 807.8 | 2221.6 | 1971.1 | 2243.1 | 2101.5 | 2608.1 | 1696.3 | 2717.8 | 3153.4 |
| MIP-1A | 319.0 | 344.0* | 56.7* | 25.8 | 9.5 | 293.3 | 231.3 | 302.3 | 405.7 | 190.8 | 85.4 | 413.5 | 682.2 |
| MIP-1B | 476.5 | 457.0 | 114.1* | 133.2 | 102.5 | 428.6 | 248.0 | 432.5 | 430.0 | 253.6 | 162.6 | 1069.9 | 506.9 |
| TNF-α | 86.0 | 72.5 | 24.4* | 32.8 | 19.96 | 74.8 | 31.9 | 75.1 | 80.8 | 40.7 | 29.0 | 92.6 | 256.8 |

Values in pg/mL
CHIKV: chikungunya virus
*$p<0.05$

**Supplementary Table S1.** Cytokine values and demographics in patients with confirmed CHIKV infection part 2.

| | Social strata | | | | | Health care | | | | |
|---|---|---|---|---|---|---|---|---|---|---|
| | Strata 1 | Strata 2 | Strata 3 | Strata 4 | Strata 5 | Taxpayer | Beneficiary | Subsidized | Special regime | Private health care |
| Eotaxin | 188.2 | 165.5 | 187.7 | 119.6 | 466.0* | 182.4 | 201.8 | 176.5 | 98.1 | 785.1 |
| FGF-2 | 79.6 | 95.7 | 83.3 | 95.4 | 123.1* | 98.6 | 80.7 | 86.9 | 71.7 | 95.4 |
| GM-CFS | 14.4 | 23.9 | 13.9 | 15.7 | 78.3 | 13.3 | 20.7 | 20.9 | 10.0 | 16.6 |
| INF-α | 37.6 | 46.6 | 44.8 | 55.6 | 68.5 | 42.2 | 44.8 | 43.6 | 38.1 | 61.9 |
| INF-γ | 14.0 | 16.2 | 16.2 | 18.2 | 22.0* | 15.8 | 16.8 | 15.1 | 7.1 | 10.6 |
| IL-1B | 26.2 | 70.0 | 20.3 | 7.5 | 2659.5 | 16.2 | 323.0 | 55.6 | 1.3 | 7.1 |
| IL-1RA | 122.3 | 744.0 | 133.8 | 81.4 | 204.6* | 159.9 | 150.9 | 484.9 | 97.6 | 106.1 |
| IL-2 | 3.1 | 3.5 | 3.3 | 2.9 | 6.8 | 3.9 | 3.4 | 3.2 | 0.9 | 2.2 |
| IL-4 | 264.0 | 156.7* | 346.6 | 96.4 | 169.1 | 297.7 | 222.5 | 220.0 | 3.2 | 570.2 |
| IL-6 | 44.7 | 144.6 | 121.1 | 13.6 | 930.1* | 51.2 | 196.1 | 110.5 | 3.2 | 54.2 |
| IL-7 | 14.3 | 16.9 | 18.8 | 14.2 | 20.3 | 17.3 | 16.3 | 16.1 | 3.2 | 21.1 |
| IL-8 | 2280.5 | 3580.2 | 4256.6 | 2333.8 | 7170.4 | 3865.7 | 4469.8 | 2681.7 | 14.8 | 271.7 |

| | | | | | | | | | | |
|---|---|---|---|---|---|---|---|---|---|---|
| IL-10 | 15.6* | 21.1 | 17.0 | 13.7 | 35.6* | 18.3 | 23.9 | 16.4 | 3.2 | 15.8 |
| IL-12p40 | 16.8 | 18.9 | 18.7 | 24.6 | 34.9* | 17.7 | 20.6 | 18.3 | 3.2 | 40.4 |
| IL-12p70 | 11.5 | 10.4 | 15.4 | 9.9 | 14.9* | 12.0 | 12.0 | 11.9 | 3.4 | 20.2 |
| IL-15 | 6.8 | 7.5 | 7.6 | 9.3 | 10.9* | 7.5 | 7.9 | 7.2 | 3.1 | 14.2 |
| IL-17A | 11.6 | 14.0 | 12.6 | 14.2 | 25.2* | 12.5 | 14.3 | 12.9 | 4.9 | 16.3 |
| IP-10 | 569.2 | 679.4 | 756.0 | 558.8 | 942.8 | 686.3 | 754.0 | 610.7 | 318.5 | 943.2 |
| MCP-1 | 1926.6 | 2263.9 | 2602.5 | 1768.3 | 3004.8 | 2598.8 | 2250.7 | 2080.5 | 393.6 | 2709.0 |
| MIP-1A | 275.7 | 309.9 | 234.2 | 51.0 | 968.8 | 142.4 | 352.2 | 318.7 | 2.4* | 104.5 |
| MIP-1B | 388.0 | 554.0 | 284.6 | 175.6 | 333.3* | 211.8 | 253.0 | 552.2 | 15.6* | 350.9 |
| TNF-α | 36.5 | 90.1 | 58.1 | 28.9 | 535.6* | 36.5 | 108.7 | 70.8 | 17.7 | 84.8 |

Values in pg/mL
CHIKV: chikungunya virus
*$p<0.05$

**Supplementary Table S2**. Point-biserial correlation analysis of cytokines in patients with confirmed CHIKV infection.

| | Eotaxin | FGF-2 | GM-CFS | INF-α | INF-γ | IL-1B | IL-1RA | IL-2 | IL-4 | IL-6 | IL-7 | IL-8 | IL-10 | IL-12p40 | IL-12p70 | IL-15 | IL-17A | IP-10 | MCP-1 | MIP-1A | MIP-1B | TNF-α |
|---|---|---|---|---|---|---|---|---|---|---|---|---|---|---|---|---|---|---|---|---|---|---|
| Eotaxin | | Pearson: 0.205 P=0.001 | Pearson: 0.082 P=0.170 | Pearson: 0.191 P=0.001 | Pearson: 0.172 P=0.004 | Pearson: 0.064 P=0.285 | Pearson: 0.064 P=0.284 | Pearson: 0.289 P=0.000 | Pearson: 0.131 P=0.028 | Pearson: 0.076 P=0.203 | Pearson: 0.238 P=0.000 | Pearson: 0.191 P=0.001 | Pearson: 0.139 P=0.002 | Pearson: 0.310 P=0.000 | Pearson: 0.141 P=0.018 | Pearson: 0.336 P=0.000 | Pearson: 0.334 P=0.000 | Pearson: 0.269 P=0.000 | Pearson: 0.315 P=0.000 | Pearson: 0.068 P=0.260 | Pearson: 0.059 P=0.326 | Pearson: 0.120 P=0.046 |
| FGF-2 | Pearson: 0.205 P=0.001 | | Pearson: 0.171 P=0.004 | Pearson: 0.487 P=0.000 | Pearson: 0.214 P=0.000 | Pearson: 0.062 P=0.306 | Pearson: 0.056 P=0.351 | Pearson: 0.154 P=0.010 | Pearson: 0.157 P=0.008 | Pearson: 0.133 P=0.026 | Pearson: 0.349 P=0.000 | Pearson: 0.278 P=0.000 | Pearson: 0.234 P=0.000 | Pearson: 0.621 P=0.000 | Pearson: 0.170 P=0.004 | Pearson: 0.553 P=0.000 | Pearson: 0.484 P=0.000 | Pearson: 0.283 P=0.000 | Pearson: 0.351 P=0.000 | Pearson: 0.098 P=0.102 | Pearson: 0.143 P=0.017 | Pearson: 0.120 P=0.046 |
| GM-CFS | Pearson: 0.082 | Pearson: 0.171 | | Pearson: 0.285 | Pearson: 0.073 | Pearson: 0.589 | Pearson: 0.036 | Pearson: 0.064 | Pearson: 0.088 | Pearson: 0.768 | Pearson: 0.231 | Pearson: 0.467 | Pearson: 0.561 | Pearson: 0.297 | Pearson: 0.082 | Pearson: 0.259 | Pearson: 0.187 | Pearson: 0.033 | Pearson: 0.262 | Pearson: 0.565 | Pearson: 0.511 | Pearson: 0.546 |

| | | | | | | | | | | | | | | | | | | | | | | |
|---|---|---|---|---|---|---|---|---|---|---|---|---|---|---|---|---|---|---|---|---|---|---|
| | P=0.170 | P=0.004 | | P=0.000 | P=0.222 | P=0.000 | P=0.551 | P=0.286 | P=0.144 | P=0.000 | P=0.000 | P=0.000 | P=0.000 | P=0.000 | P=0.171 | P=0.000 | P=0.002 | P=0.580 | P=0.000 | P=0.000 | P=0.000 | P=0.000 |
| INF-α | Pearson: 0.191 P=0.001 | Pearson: 0.487 P=0.000 | Pearson: 0.285 P=0.000 | | Pearson: 0.185 P=0.002 | Pearson: 0.120 P=0.045 | Pearson: 0.067 P=0.262 | Pearson: 0.119 P=0.048 | Pearson: 0.092 P=0.127 | Pearson: 0.220 P=0.000 | Pearson: 0.474 P=0.000 | Pearson: 0.388 P=0.000 | Pearson: 0.364 P=0.000 | Pearson: 0.732 P=0.000 | Pearson: 0.249 P=0.000 | Pearson: 0.735 P=0.000 | Pearson: 0.606 P=0.000 | Pearson: 0.388 P=0.000 | Pearson: 0.473 P=0.000 | Pearson: 0.215 P=0.000 | Pearson: 0.216 P=0.000 | Pearson: 0.197 P=0.000 |
| INF-γ | Pearson: 0.172 P=0.004 | Pearson: 0.214 P=0.000 | Pearson: 0.073 P=0.222 | Pearson: 0.185 P=0.002 | | Pearson: 0.019 P=0.755 | Pearson: 0.010 P=0.866 | Pearson: 0.314 P=0.000 | Pearson: 0.092 P=0.124 | Pearson: 0.046 P=0.448 | Pearson: 0.126 P=0.035 | Pearson: 0.052 P=0.387 | Pearson: 0.136 P=0.023 | Pearson: 0.222 P=0.000 | Pearson: 0.247 P=0.000 | Pearson: 0.251 P=0.000 | Pearson: 0.538 P=0.000 | Pearson: 0.271 P=0.000 | Pearson: 0.124 P=0.039 | Pearson: 0.018 P=0.763 | Pearson: 0.056 P=0.355 | Pearson: 0.054 P=0.366 |
| IL-1B | Pearson: 0.064 P=0.285 | Pearson: 0.062 P=0.306 | Pearson: 0.589 P=0.000 | Pearson: 0.120 P=0.045 | Pearson: 0.019 P=0.755 | | Pearson: 0.013 P=0.823 | Pearson: 0.034 P=0.577 | Pearson: 0.008 P=0.892 | Pearson: 0.739 P=0.000 | Pearson: 0.097 P=0.106 | Pearson: 0.392 P=0.000 | Pearson: 0.352 P=0.000 | Pearson: 0.139 P=0.020 | Pearson: 0.026 P=0.666 | Pearson: 0.132 P=0.028 | Pearson: 0.076 P=0.209 | Pearson: 0.036 P=0.546 | Pearson: 0.183 P=0.002 | Pearson: 0.474 P=0.000 | Pearson: 0.150 P=0.012 | Pearson: 0.695 P=0.000 |
| IL-1RA | Pearson: 0.064 P=0.284 | Pearson: 0.056 P=0.351 | Pearson: 0.036 P=0.551 | Pearson: 0.067 P=0.262 | Pearson: 0.010 P=0.866 | Pearson: 0.013 P=0.823 | | Pearson: 0.044 P=0.459 | Pearson: 0.013 P=0.835 | Pearson: 0.024 P=0.693 | Pearson: 0.018 P=0.763 | Pearson: 0.019 P=0.749 | Pearson: 0.039 P=0.521 | Pearson: 0.044 P=0.466 | Pearson: 0.015 P=0.808 | Pearson: 0.039 P=0.514 | Pearson: 0.102 P=0.088 | Pearson: 0.009 P=0.884 | Pearson: 0.016 P=0.784 | Pearson: 0.013 P=0.826 | Pearson: 0.021 P=0.733 | Pearson: 0.011 P=0.855 |
| IL-2 | Pearson: 0.289 P=0.000 | Pearson: 0.154 P=0.010 | Pearson: 0.064 P=0.286 | Pearson: 0.119 P=0.048 | Pearson: 0.314 P=0.000 | Pearson: 0.034 P=0.577 | Pearson: 0.044 P=0.459 | | Pearson: 0.054 P=0.373 | Pearson: 0.046 P=0.442 | Pearson: 0.225 P=0.000 | Pearson: 0.055 P=0.356 | Pearson: 0.050 P=0.402 | Pearson: 0.124 P=0.039 | Pearson: 0.119 P=0.046 | Pearson: 0.159 P=0.008 | Pearson: 0.255 P=0.000 | Pearson: 0.238 P=0.000 | Pearson: 0.094 P=0.117 | Pearson: 0.025 P=0.681 | Pearson: 0.032 P=0.592 | Pearson: 0.047 P=0.439 |
| IL-4 | Pearson: 0.131 P=0.028 | Pearson: 0.157 P=0.008 | Pearson: 0.088 P=0.144 | Pearson: 0.092 P=0.127 | Pearson: 0.092 P=0.124 | Pearson: 0.008 P=0.892 | Pearson: 0.013 P=0.835 | Pearson: 0.054 P=0.373 | | Pearson: 0.174 P=0.004 | Pearson: 0.016 P=0.787 | Pearson: 0.070 P=0.246 | Pearson: 0.007 P=0.907 | Pearson: 0.250 P=0.000 | Pearson: 0.062 P=0.298 | Pearson: 0.256 P=0.000 | Pearson: 0.114 P=0.057 | Pearson: 0.097 P=0.107 | Pearson: 0.070 P=0.243 | Pearson: 0.113 P=0.059 | Pearson: 0.227 P=0.000 | Pearson: 0.271 P=0.000 |
| IL-6 | Pearson: 0.076 | Pearson: 0.133 | Pearson: 0.768 | Pearson: 0.220 | Pearson: 0.046 | Pearson: 0.739 | Pearson: 0.024 | Pearson: 0.046 | Pearson: 0.174 | | Pearson: 0.234 | Pearson: 0.585 | Pearson: 0.540 | Pearson: 0.257 | Pearson: 0.041 | Pearson: 0.240 | Pearson: 0.131 | Pearson: 0.000 | Pearson: 0.322 | Pearson: 0.821 | Pearson: 0.521 | Pearson: 0.856 |

| | | | | | | | | | | | | | | | | | | | | | | |
|---|---|---|---|---|---|---|---|---|---|---|---|---|---|---|---|---|---|---|---|---|---|---|
| | P=0.203 | P=0.026 | P=0.000 | P=0.000 | P=0.448 | P=0.000 | P=0.693 | P=0.442 | P=0.004 | | P=0.000 | P=0.000 | P=0.000 | P=0.000 | P=0.495 | P=0.000 | P=0.028 | P=0.999 | P=0.000 | P=0.000 | P=0.000 | P=0.000 |
| IL-7 | Pearson: 0.238 P=0.000 | Pearson: 0.349 P=0.000 | Pearson: 0.231 P=0.000 | Pearson: 0.474 P=0.000 | Pearson: 0.126 P=0.035 | Pearson: 0.097 P=0.106 | Pearson: 0.018 P=0.763 | Pearson: 0.225 P=0.000 | Pearson: 0.016 P=0.787 | Pearson: 0.234 P=0.000 | | Pearson: 0.338 P=0.000 | Pearson: 0.293 P=0.000 | Pearson: 0.430 P=0.000 | Pearson: 0.141 P=0.019 | Pearson: 0.485 P=0.000 | Pearson: 0.430 P=0.000 | Pearson: 0.193 P=0.001 | Pearson: 0.379 P=0.000 | Pearson: 0.212 P=0.000 | Pearson: 0.185 P=0.002 | Pearson: 0.176 P=0.003 |
| IL-8 | Pearson: 0.191 P=0.001 | Pearson: 0.278 P=0.000 | Pearson: 0.467 P=0.000 | Pearson: 0.388 P=0.000 | Pearson: 0.052 P=0.387 | Pearson: 0.392 P=0.000 | Pearson: 0.019 P=0.749 | Pearson: 0.055 P=0.356 | Pearson: 0.070 P=0.246 | Pearson: 0.585 P=0.000 | Pearson: 0.338 P=0.000 | | Pearson: 0.556 P=0.000 | Pearson: 0.446 P=0.000 | Pearson: 0.073 P=0.224 | Pearson: 0.472 P=0.000 | Pearson: 0.228 P=0.000 | Pearson: 0.177 P=0.003 | Pearson: 0.726 P=0.000 | Pearson: 0.624 P=0.000 | Pearson: 0.340 P=0.000 | Pearson: 0.519 P=0.000 |
| IL-10 | Pearson: 0.139 P=0.002 | Pearson: 0.234 P=0.000 | Pearson: 0.561 P=0.000 | Pearson: 0.364 P=0.000 | Pearson: 0.136 P=0.023 | Pearson: 0.352 P=0.000 | Pearson: 0.039 P=0.521 | Pearson: 0.050 P=0.402 | Pearson: 0.007 P=0.907 | Pearson: 0.540 P=0.000 | Pearson: 0.293 P=0.000 | Pearson: 0.556 P=0.000 | | Pearson: 0.461 P=0.000 | Pearson: 0.162 P=0.007 | Pearson: 0.358 P=0.000 | Pearson: 0.377 P=0.000 | Pearson: 0.213 P=0.000 | Pearson: 0.518 P=0.000 | Pearson: 0.483 P=0.000 | Pearson: 0.302 P=0.000 | Pearson: 0.327 P=0.000 |
| IL-12p40 | Pearson: 0.310 P=0.000 | Pearson: 0.621 P=0.000 | Pearson: 0.297 P=0.000 | Pearson: 0.732 P=0.000 | Pearson: 0.222 P=0.000 | Pearson: 0.139 P=0.020 | Pearson: 0.044 P=0.466 | Pearson: 0.124 P=0.039 | Pearson: 0.250 P=0.000 | Pearson: 0.257 P=0.000 | Pearson: 0.430 P=0.000 | Pearson: 0.446 P=0.000 | Pearson: 0.461 P=0.000 | | Pearson: 0.234 P=0.000 | Pearson: 0.856 P=0.000 | Pearson: 0.657 P=0.000 | Pearson: 0.423 P=0.000 | Pearson: 0.575 P=0.000 | Pearson: 0.254 P=0.000 | Pearson: 0.248 P=0.000 | Pearson: 0.262 P=0.000 |
| IL-12p70 | Pearson: 0.141 P=0.018 | Pearson: 0.170 P=0.004 | Pearson: 0.082 P=0.171 | Pearson: 0.249 P=0.000 | Pearson: 0.247 P=0.000 | Pearson: 0.026 P=0.666 | Pearson: 0.015 P=0.808 | Pearson: 0.119 P=0.046 | Pearson: 0.062 P=0.298 | Pearson: 0.041 P=0.495 | Pearson: 0.141 P=0.019 | Pearson: 0.073 P=0.224 | Pearson: 0.162 P=0.007 | Pearson: 0.234 P=0.000 | | Pearson: 0.206 P=0.001 | Pearson: 0.375 P=0.000 | Pearson: 0.208 P=0.000 | Pearson: 0.160 P=0.008 | Pearson: 0.026 P=0.661 | Pearson: 0.041 P=0.491 | Pearson: 0.052 P=0.279 |
| IL-15 | Pearson: 0.336 P=0.000 | Pearson: 0.553 P=0.000 | Pearson: 0.259 P=0.000 | Pearson: 0.735 P=0.000 | Pearson: 0.251 P=0.000 | Pearson: 0.132 P=0.028 | Pearson: 0.039 P=0.514 | Pearson: 0.159 P=0.008 | Pearson: 0.256 P=0.000 | Pearson: 0.240 P=0.000 | Pearson: 0.485 P=0.000 | Pearson: 0.472 P=0.000 | Pearson: 0.358 P=0.000 | Pearson: 0.856 P=0.000 | Pearson: 0.206 P=0.001 | | Pearson: 0.613 P=0.000 | Pearson: 0.448 P=0.000 | Pearson: 0.616 P=0.000 | Pearson: 0.226 P=0.000 | Pearson: 0.211 P=0.000 | Pearson: 0.229 P=0.000 |
| IL-17A | Pearson: 0.334 | Pearson: 0.484 | Pearson: 0.187 | Pearson: 0.606 | Pearson: 0.538 | Pearson: 0.076 | Pearson: 0.102 | Pearson: 0.255 | Pearson: 0.114 | Pearson: 0.131 | Pearson: 0.430 | Pearson: 0.228 | Pearson: 0.377 | Pearson: 0.657 | Pearson: 0.375 | Pearson: 0.613 | | Pearson: 0.265 | Pearson: 0.381 | Pearson: 0.117 | Pearson: 0.144 | Pearson: 0.128 |

| | | | | | | | | | | | | | | | | | | | | | | |
|---|---|---|---|---|---|---|---|---|---|---|---|---|---|---|---|---|---|---|---|---|---|---|
| | P=0.000 | P=0.000 | P=0.002 | P=0.000 | P=0.000 | P=0.209 | P=0.088 | P=0.000 | P=0.057 | P=0.028 | P=0.000 | P=0.000 | P=0.000 | P=0.000 | P=0.000 | P=0.000 | | P=0.000 | P=0.000 | P=0.050 | P=0.016 | P=0.000 |
| IP-10 | Pearson: 0.269 P=0.000 | Pearson: 0.283 P=0.000 | Pearson: 0.033 P=0.580 | Pearson: 0.388 P=0.000 | Pearson: 0.271 P=0.000 | Pearson: 0.036 P=0.546 | Pearson: 0.009 P=0.884 | Pearson: 0.238 P=0.000 | Pearson: 0.097 P=0.107 | Pearson: 0.000 P=0.999 | Pearson: 0.193 P=0.001 | Pearson: 0.177 P=0.003 | Pearson: 0.213 P=0.000 | Pearson: 0.423 P=0.000 | Pearson: 0.208 P=0.000 | Pearson: 0.448 P=0.000 | Pearson: 0.265 P=0.000 | | Pearson: 0.317 P=0.000 | Pearson: 0.012 P=0.841 | Pearson: 0.002 P=0.970 | Pearson: 0.010 P=0.874 |
| MCP-1 | Pearson: 0.315 P=0.000 | Pearson: 0.351 P=0.000 | Pearson: 0.262 P=0.000 | Pearson: 0.473 P=0.000 | Pearson: 0.124 P=0.039 | Pearson: 0.183 P=0.002 | Pearson: 0.016 P=0.784 | Pearson: 0.094 P=0.117 | Pearson: 0.070 P=0.243 | Pearson: 0.322 P=0.000 | Pearson: 0.379 P=0.000 | Pearson: 0.726 P=0.000 | Pearson: 0.518 P=0.000 | Pearson: 0.575 P=0.000 | Pearson: 0.160 P=0.008 | Pearson: 0.616 P=0.000 | Pearson: 0.117 P=0.050 | Pearson: 0.317 P=0.000 | | Pearson: 0.360 P=0.000 | Pearson: 0.263 P=0.000 | Pearson: 0.318 P=0.000 |
| MIP-1A | Pearson: 0.068 P=0.260 | Pearson: 0.098 P=0.102 | Pearson: 0.565 P=0.000 | Pearson: 0.215 P=0.000 | Pearson: 0.018 P=0.763 | Pearson: 0.474 P=0.000 | Pearson: 0.013 P=0.826 | Pearson: 0.025 P=0.681 | Pearson: 0.113 P=0.059 | Pearson: 0.821 P=0.000 | Pearson: 0.212 P=0.000 | Pearson: 0.624 P=0.000 | Pearson: 0.483 P=0.000 | Pearson: 0.254 P=0.000 | Pearson: 0.026 P=0.661 | Pearson: 0.226 P=0.000 | Pearson: 0.117 P=0.050 | Pearson: 0.012 P=0.841 | Pearson: 0.360 P=0.000 | | Pearson: 0.506 P=0.000 | Pearson: 0.706 P=0.000 |
| MIP-1B | Pearson: 0.059 P=0.326 | Pearson: 0.143 P=0.017 | Pearson: 0.511 P=0.000 | Pearson: 0.216 P=0.000 | Pearson: 0.056 P=0.355 | Pearson: 0.150 P=0.012 | Pearson: 0.021 P=0.733 | Pearson: 0.032 P=0.592 | Pearson: 0.227 P=0.000 | Pearson: 0.521 P=0.000 | Pearson: 0.185 P=0.002 | Pearson: 0.340 P=0.000 | Pearson: 0.302 P=0.000 | Pearson: 0.248 P=0.000 | Pearson: 0.041 P=0.491 | Pearson: 0.211 P=0.000 | Pearson: 0.144 P=0.016 | Pearson: 0.002 P=0.970 | Pearson: 0.263 P=0.000 | Pearson: 0.506 P=0.000 | | Pearson: 0.574 P=0.000 |
| TNF-α | Pearson: 0.120 P=0.046 | Pearson: 0.120 P=0.046 | Pearson: 0.546 P=0.000 | Pearson: 0.197 P=0.000 | Pearson: 0.054 P=0.366 | Pearson: 0.695 P=0.000 | Pearson: 0.011 P=0.855 | Pearson: 0.047 P=0.439 | Pearson: 0.271 P=0.000 | Pearson: 0.856 P=0.000 | Pearson: 0.176 P=0.003 | Pearson: 0.519 P=0.000 | Pearson: 0.327 P=0.000 | Pearson: 0.262 P=0.000 | Pearson: 0.052 P=0.279 | Pearson: 0.229 P=0.000 | Pearson: 0.128 P=0.000 | Pearson: 0.010 P=0.874 | Pearson: 0.318 P=0.000 | Pearson: 0.706 P=0.000 | Pearson: 0.574 P=0.000 | |

CHIKV: chikungunya virus; Green: strong correlation; Yellow: moderate correlation; Red: weak correlation

**Supplementary Table S3.** Point-biserial correlation analysis of demographics and cytokines in patients with confirmed CHIKV infection part 1.

| | Ethnicity | | | | | Literacy | | Educational level | | | | | |
|---|---|---|---|---|---|---|---|---|---|---|---|---|---|
| | Mestizo | Caucasian | Afro-American | Indigenous | Other | Read & writes | Illiteracy | Some education | Primary school | Middle school | High school | Bachelor | University |
| Eotaxin | Pearson: - 0.033 p=0.588 | Pearson: 0.037 p=0.540 | Pearson: 0.051 p=0.339 | Pearson: 0.115 p=0.055 | Pearson: - 0.052 p=0.386 | Pearson: 0.051 p=0.395 | Pearson: - 0.051 p=0.395 | Pearson: 0.060 p=0.318 | Pearson: - 0.005 p=0.931 | Pearson: - 0.012 p=0.837 | Pearson: - 0.109 p=0.069 | Pearson: 0.178 P=0.003 | Pearson: 0.031 p=0.604 |
| FGF-2 | Pearson: - 0.037 p=0.540 | Pearson: 0.151 P=0.012 | Pearson: - 0.146 P=0.015 | Pearson: - 0.039 p=0.516 | Pearson: - 0.036 p=0.549 | Pearson: 0.004 p=0.947 | Pearson: - 0.004 p=0.947 | Pearson: - 0.032 p=0.590 | Pearson: 0.037 p=0.543 | Pearson: 0.028 p=0.643 | Pearson: - 0.073 p=0.227 | Pearson: - 0.029 p=0.631 | Pearson: 0.024 p=0.693 |
| GM-CFS | Pearson: 0.038 p=0.528 | Pearson: 0.007 p=0.902 | Pearson: - 0.056 p=0.355 | Pearson: - 0.025 p=0.681 | Pearson: - 0.014 p=0.814 | Pearson: 0.008 p=0.893 | Pearson: - 0.008 p=0.893 | Pearson: 0.018 p=0.762 | Pearson: - 0.029 p=0.624 | Pearson: - 0.028 p=0.638 | Pearson: - 0.065 p=0.277 | Pearson: 0.109 p=0.069 | Pearson: 0.072 p=0.228 |
| INF-$\alpha$ | Pearson: - 0.008 p=0.893 | Pearson: 0.088 p=0.143 | Pearson: - 0.147 P=0.014 | Pearson: 0.043 p=0.476 | Pearson: - 0.008 p=0.896 | Pearson: - 0.007 p=0.906 | Pearson: 0.007 p=0.906 | Pearson: - 0.015 p=0.803 | Pearson: - 0.032 p=0.595 | Pearson: 0.040 p=0.501 | Pearson: - 0.051 p=0.396 | Pearson: - 0.003 p=0.960 | Pearson: 0.084 p=0.163 |
| INF-$\gamma$ | Pearson: 0.113 p=0.061 | Pearson: - 0.015 p=0.809 | Pearson: - 0.147 P=0.014 | Pearson: 0.003 p=0.965 | Pearson: - 0.046 p=0.448 | Pearson: 0.035 p=0.559 | Pearson: - 0.035 p=0.559 | Pearson: - 0.016 p=0.795 | Pearson: - 0.056 p=0.353 | Pearson: 0.044 p=0.467 | Pearson: 0.042 p=0.482 | Pearson: - 0.047 p=0.434 | Pearson: 0.019 p=0.746 |
| IL-1B | Pearson: - 0.038 p=0.527 | Pearson: 0.065 p=0.281 | Pearson: - 0.030 p=0.616 | Pearson: - 0.014 p=0.822 | Pearson: - 0.009 p=0.876 | Pearson: 0.020 p=0.738 | Pearson: - 0.020 p=0.738 | Pearson: 0.022 p=0.713 | Pearson: - 0.040 p=0.501 | Pearson: - 0.031 p=0.608 | Pearson: - 0.049 p=0.417 | Pearson: 0.006 p=0.923 | Pearson: 0.213 P=0.000 |
| IL-1RA | Pearson: - 0.075 p=0.214 | Pearson: 0.108 p=0.071 | Pearson: - 0.036 p=0.549 | Pearson: - 0.018 p=0.761 | Pearson: - 0.013 p=0.835 | Pearson: - 0.153 P=0.011 | Pearson: 0.153 P=0.011 | Pearson: - 0.140 P=0.019 | Pearson: - 0.057 p=0.339 | Pearson: - 0.035 p=0.563 | Pearson: 0.041 p=0.492 | Pearson: - 0.031 p=0.600 | Pearson: - 0.015 p=0.799 |
| IL-2 | Pearson: - 0.037 | Pearson: 0.098 | Pearson: - 0.053 | Pearson: - 0.061 | Pearson: - 0.027 | Pearson: 0.020 | Pearson: - 0.020 | Pearson: 0.130 | Pearson: - 0.058 | Pearson: - 0.031 | Pearson: - 0.039 | Pearson: 0.103 | Pearson: - 0.061 |

| | | | | | | | | | | | | | |
|---|---|---|---|---|---|---|---|---|---|---|---|---|---|
| | p=0.539 | p=0.104 | p=0.382 | p=0.312 | p=0.655 | p=0.735 | p=0.735 | P=0.030 | p=0.332 | p=0.612 | p=0.519 | p=0.087 | p=0.313 |
| IL-4 | Pearson: 0.120 P=0.045 | Pearson: -0.124 P=0.039 | Pearson: 0.027 p=0.655 | Pearson: -0.042 p=0.489 | Pearson: -0.011 p=0.861 | Pearson: 0.035 p=0.558 | Pearson: -0.035 p=0.558 | Pearson: 0.027 p=0.651 | Pearson: 0.037 p=0.535 | Pearson: 0.001 p=0.993 | Pearson: -0.054 p=0.369 | Pearson: 0.055 p=0.364 | Pearson: -0.014 p=0.815 |
| IL-6 | Pearson: 0.059 p=0.322 | Pearson: -0.011 p=0.852 | Pearson: -0.057 p=0.342 | Pearson: -0.030 p=0.614 | Pearson: -0.017 p=0.777 | Pearson: 0.030 p=0.622 | Pearson: -0.030 p=0.622 | Pearson: 0.029 p=0.626 | Pearson: -0.036 p=0.551 | Pearson: -0.038 p=0.525 | Pearson: -0.095 p=0.115 | Pearson: 0.097 p=0.106 | Pearson: 0.177 P=0.003 |
| IL-7 | Pearson: -0.019 p=0.749 | Pearson: 0.101 p=0.091 | Pearson: -0.105 p=0.081 | Pearson: -0.034 p=0.573 | Pearson: -0.020 p=0.745 | Pearson: 0.064 p=0.279 | Pearson: -0.064 p=0.288 | Pearson: 0.066 p=0.272 | Pearson: -0.058 p=0.333 | Pearson: 0.042 p=0.486 | Pearson: -0.122 P=0.042 | Pearson: 0.166 P=0.005 | Pearson: 0.094 p=0.117 |
| IL-8 | Pearson: -0.045 p=0.452 | Pearson: 0.146 P=0.014 | Pearson: -0.116 p=0.053 | Pearson: -0.052 p=0.388 | Pearson: -0.042 p=0.487 | Pearson: 0.058 p=0.330 | Pearson: -0.058 p=0.330 | Pearson: 0.066 p=0.272 | Pearson: 0.026 p=0.660 | Pearson: 0.084 p=0.160 | Pearson: -0.110 p=0.066 | Pearson: -0.016 p=0.786 | Pearson: 0.123 P=0.041 |
| IL-10 | Pearson: 0.049 p=0.412 | Pearson: 0.039 p=0.516 | Pearson: -0.108 p=0.073 | Pearson: -0.045 p=0.459 | Pearson: -0.032 p=0.595 | Pearson: 0.006 p=0.919 | Pearson: -0.006 p=0.919 | Pearson: 0.014 p=0.815 | Pearson: -0.010 p=0.864 | Pearson: 0.032 p=0.599 | Pearson: -0.093 p=0.122 | Pearson: 0.042 p=0.481 | Pearson: 0.096 p=0.109 |
| IL-12p40 | Pearson: 0.003 p=0.958 | Pearson: 0.113 p=0.060 | Pearson: -0.147 P=0.014 | Pearson: -0.069 p=0.249 | Pearson: 0.000 p=0.996 | Pearson: -0.017 p=0.775 | Pearson: 0.017 p=0.775 | Pearson: 0.058 p=0.331 | Pearson: 0.035 p=0.555 | Pearson: 0.051 p=0.400 | Pearson: 0.089 p=0.137 | Pearson: 0.003 p=0.966 | Pearson: 0.075 p=0.214 |
| IL-12p70 | Pearson: -0.022 p=0.718 | Pearson: 0.009 p=0.879 | Pearson: 0.016 p=0.790 | Pearson: 0.026 p=0.660 | Pearson: -0.028 p=0.639 | Pearson: 0.026 p=0.670 | Pearson: -0.026 p=0.670 | Pearson: -0.030 p=0.618 | Pearson: 0.028 p=0.644 | Pearson: -0.099 p=0.887 | Pearson: -0.076 p=0.209 | Pearson: 0.065 p=0.280 | Pearson: -0.023 p=0.699 |
| IL-15 | Pearson: 0.032 p=0.594 | Pearson: 0.081 p=0.176 | Pearson: -0.152 P=0.011 | Pearson: -0.042 p=0.485 | Pearson: -0.020 p=0.740 | Pearson: 0.064 p=0.290 | Pearson: -0.064 p=0.290 | Pearson: 0.094 p=0.118 | Pearson: -0.019 p=0.758 | Pearson: 0.104 p=0.082 | Pearson: -0.091 p=0.129 | Pearson: 0.030 p=0.620 | Pearson: 0.112 p=0.062 |
| IL-17A | Pearson: 0.051 p=0.398 | Pearson: 0.038 p=0.523 | Pearson: -0.137 P=0.022 | Pearson: -0.001 p=0.990 | Pearson: -0.020 p=0.743 | Pearson: 0.000 p=0.995 | Pearson: 0.000 p=0.995 | Pearson: 0.008 p=0.892 | Pearson: 0.017 p=0.772 | Pearson: 0.022 p=0.718 | Pearson: -0.049 p=0.410 | Pearson: -0.002 p=0.979 | Pearson: 0.040 p=0.510 |

| | | | | | | | | | | | | | |
|---|---|---|---|---|---|---|---|---|---|---|---|---|---|
| IP-10 | Pearson: 0.074 p=0.220 | Pearson: -0.002 p=0.976 | Pearson: -0.099 p=0.098 | Pearson: 0.014 p=0.819 | Pearson: -0.077 p=0.200 | Pearson: 0.028 p=0.645 | Pearson: -0.028 p=0.645 | Pearson: 0.041 p=0.499 | Pearson: 0.009 p=0.875 | Pearson: 0.124 P=0.039 | Pearson: -0.100 p=0.094 | Pearson: 0.051 p=0.401 | Pearson: -0.037 p=0.534 |
| MCP-1 | Pearson: -0.034 p=0.575 | Pearson: 0.155 P=0.009 | Pearson: -0.143 P=0.017 | Pearson: -0.059 p=0.326 | Pearson: -0.049 p=0.411 | Pearson: 0.020 p=0.736 | Pearson: -0.020 p=0.736 | Pearson: 0.045 p=0.455 | Pearson: -0.023 p=0.701 | Pearson: 0.056 p=0.348 | Pearson: -0.102 p=0.090 | Pearson: 0.072 p=0.231 | Pearson: 0.089 p=0.138 |
| MIP-1A | Pearson: 0.027 p=0.657 | Pearson: 0.040 p=0.501 | Pearson: -0.075 p=0.210 | Pearson: -0.043 p=0.475 | Pearson: -0.028 p=0.645 | Pearson: 0.014 p=0.815 | Pearson: -0.014 p=0.815 | Pearson: 0.044 p=0.467 | Pearson: 0.072 p=0.232 | Pearson: -0.039 p=0.518 | Pearson: 0.114 p=0.057 | Pearson: 0.049 p=0.417 | Pearson: 0.104 p=0.084 |
| MIP-1B | Pearson: 0.030 p=0.619 | Pearson: 0.016 p=0.785 | Pearson: -0.055 p=0.361 | Pearson: -0.026 p=0.666 | Pearson: -0.017 p=0.771 | Pearson: 0.023 p=0.703 | Pearson: -0.023 p=0.703 | Pearson: 0.029 p=0.633 | Pearson: 0.004 p=0.943 | Pearson: -0.036 p=0.547 | Pearson: -0.080 p=0.183 | Pearson: 0.144 P=0.016 | Pearson: 0.013 p=0.826 |
| TNF-α | Pearson: 0.042 p=0.488 | Pearson: 0.001 p=0.993 | Pearson: -0.052 p=0.391 | Pearson: -0.021 p=0.721 | Pearson: -0.017 p=0.774 | Pearson: 0.033 p=0.588 | Pearson: -0.033 p=0.588 | Pearson: 0.033 p=0.580 | Pearson: 0.018 p=0.768 | Pearson: -0.041 p=0.490 | Pearson: -0.081 p=0.178 | Pearson: 0.027 p=0.656 | Pearson: 0.162 P=0.007 |

CHIKV: chikungunya virus; Green: strong correlation; Yellow: moderate correlation; Red: weak correlation

**Supplementary Table S3**. Point-biserial correlation analysis of demographics and cytokines in patients with confirmed CHIKV infection part 2.

| | Social strata | | | | | Health care | | | | |
|---|---|---|---|---|---|---|---|---|---|---|
| | Strata 1 | Strata 2 | Strata 3 | Strata 4 | Strata 5 | Taxpayer | Beneficiary | Subsidized | Special regime | Private health care |
| Eotaxin | Pearson: 0.018 P=0.760 | Pearson: -0.098 P=0.104 | Pearson: 0.010 P=0.868 | Pearson: -0.081 P=0.176 | Pearson: 0.308 P=0.000 | Pearson: -0.008 P=0.895 | Pearson: 0.061 P=0.307 | Pearson: -0.066 P=0.270 | Pearson: -0.050 P=0.403 | Pearson: 0.246 P=0.000 |
| FGF-2 | Pearson: -0.093 P=0.121 | Pearson: 0.087 P=0.148 | Pearson: -0.032 P=0.594 | Pearson: 0.021 P=0.731 | Pearson: 0.083 P=0.166 | Pearson: 0.077 P=0.198 | Pearson: -0.054 P=0.370 | Pearson: -0.014 P=0.819 | Pearson: -0.020 P=0.740 | Pearson: 0.007 P=0.910 |
| GM-CFS | Pearson: -0.058 P=0.337 | Pearson: 0.051 P=0.399 | Pearson: -0.041 P=0.492 | Pearson: -0.010 P=0.871 | Pearson: 0.142 P=0.018 | Pearson: -0.044 P=0.469 | Pearson: 0.011 P=0.849 | Pearson: 0.027 P=0.649 | Pearson: -0.012 P=0.845 | Pearson: -0.002 P=0.967 |

| | | | | | | | | | | |
|---|---|---|---|---|---|---|---|---|---|---|
| INF-α | Pearson: -0.114<br>P=0.056 | Pearson: 0.053<br>P=0.374 | Pearson: 0.014<br>P=0.811 | Pearson: 0.052<br>P=0.384 | Pearson: 0.096<br>P=0.110 | Pearson: -0.016<br>P=0.784 | Pearson: 0.015<br>P=0.801 | Pearson: -0.001<br>P=0.988 | Pearson: -0.011<br>P=0.853 | Pearson: 0.026<br>P=0.662 |
| INF-γ | Pearson: -0.069<br>P=0.254 | Pearson: 0.027<br>P=0.654 | Pearson: 0.017<br>P=0.772 | Pearson: 0.026<br>P=0.663 | Pearson: 0.056<br>P=0.348 | Pearson: 0.008<br>P=0.898 | Pearson: 0.037<br>P=0.539 | Pearson: -0.028<br>P=0.636 | Pearson: -0.039<br>P=0.516 | Pearson: -0.016<br>P=0.787 |
| IL-1B | Pearson: -0.055<br>P=0.358 | Pearson: -0.023<br>P=0.696 | Pearson: -0.038<br>P=0.525 | Pearson: -0.016<br>P=0.793 | Pearson: 0.360<br>P=0.000 | Pearson: -0.038<br>P=0.525 | Pearson: 0.101<br>P=0.093 | Pearson: -0.052<br>P=0.386 | Pearson: -0.008<br>P=0.897 | Pearson: -0.005<br>P=0.931 |
| IL-1RA | Pearson: -0.071<br>P=0.236 | Pearson: 0.119<br>P=0.047 | Pearson: -0.043<br>P=0.471 | Pearson: -0.019<br>P=0.746 | Pearson: -0.009<br>P=0.879 | Pearson: -0.036<br>P=0.546 | Pearson: -0.042<br>P=0.489 | Pearson: 0.066<br>P=0.273 | Pearson: -0.009<br>P=0.887 | Pearson: -0.006<br>P=0.923 |
| IL-2 | Pearson: -0.063<br>P=0.291 | Pearson: 0.022<br>P=0.715 | Pearson: -0.009<br>P=0.875 | Pearson: -0.029<br>P=0.629 | Pearson: 0.187<br>P=0.002 | Pearson: 0.091<br>P=0.129 | Pearson: 0.007<br>P=0.913 | Pearson: -0.063<br>P=0.296 | Pearson: -0.073<br>P=0.227 | Pearson: -0.023<br>P=0.701 |
| IL-4 | Pearson: 0.037<br>P=0.537 | Pearson: -0.094<br>P=0.116 | Pearson: 0.092<br>P=0.126 | Pearson: -0.041<br>P=0.494 | Pearson: -0.017<br>P=0.775 | Pearson: 0.049<br>P=0.412 | Pearson: -0.011<br>P=0.858 | Pearson: -0.029<br>P=0.633 | Pearson: -0.032<br>P=0.595 | Pearson: 0.033<br>P=0.587 |
| IL-6 | Pearson: -0.093<br>P=0.122 | Pearson: 0.033<br>P=0.579 | Pearson: 0.003<br>P=0.955 | Pearson: -0.031<br>P=0.609 | Pearson: 0.212<br>P=0.000 | Pearson: -0.052<br>P=0.388 | Pearson: 0.068<br>P=0.257 | Pearson: -0.012<br>P=0.837 | Pearson: -0.016<br>P=0.793 | Pearson: -0.006<br>P=0.919 |
| IL-7 | Pearson: -0.089<br>P=0.137 | Pearson: 0.026<br>P=0.664 | Pearson: 0.072<br>P=0.232 | Pearson: -0.021<br>P=0.724 | Pearson: 0.036<br>P=0.547 | Pearson: 0.027<br>P=0.651 | Pearson: -0.001<br>P=0.980 | Pearson: -0.012<br>P=0.847 | Pearson: -0.063<br>P=0.297 | Pearson: 0.016<br>P=0.790 |
| IL-8 | Pearson: -0.096<br>P=0.110 | Pearson: 0.028<br>P=0.638 | Pearson: 0.062<br>P=0.306 | Pearson: -0.021<br>P=0.727 | Pearson: 0.077<br>P=0.200 | Pearson: 0.036<br>P=0.555 | Pearson: 0.078<br>P=0.193 | Pearson: -0.085<br>P=0.156 | Pearson: -0.034<br>P=0.571 | Pearson: -0.022<br>P=0.713 |
| IL-10 | Pearson: -0.060<br>P=0.318 | Pearson: 0.059<br>P=0.322 | Pearson: -0.018<br>P=0.761 | Pearson: -0.024<br>P=0.687 | Pearson: 0.079<br>P=0.187 | Pearson: 0.000<br>P=0.995 | Pearson: 0.084<br>P=0.163 | Pearson: -0.063<br>P=0.294 | Pearson: -0.037<br>P=0.541 | Pearson: -0.004<br>P=0.943 |
| IL-12p40 | Pearson: -0.080<br>P=0.184 | Pearson: 0.012<br>P=0.846 | Pearson: 0.000<br>P=1.000 | Pearson: 0.061<br>P=0.314 | Pearson: 0.144<br>P=0.016 | Pearson: -0.027<br>P=0.655 | Pearson: 0.057<br>P=0.345 | Pearson: -0.022<br>P=0.710 | Pearson: -0.073<br>P=0.224 | Pearson: 0.072<br>P=0.230 |
| IL-12p70 | Pearson: -0.018<br>P=0.764 | Pearson: -0.062<br>P=0.305 | Pearson: 0.094<br>P=0.118 | Pearson: -0.020<br>P=0.741 | Pearson: 0.026<br>P=0.670 | Pearson: 0.001<br>P=0.991 | Pearson: 0.002<br>P=0.967 | Pearson: 0.001<br>P=0.988 | Pearson: -0.039<br>P=0.515 | Pearson: 0.027<br>P=0.658 |
| IL-15 | Pearson: -0.083<br>P=0.168 | Pearson: 0.011<br>P=0.858 | Pearson: 0.017<br>P=0.774 | Pearson: 0.065<br>P=0.280 | Pearson: 0.107<br>P=0.074 | Pearson: 0.004<br>P=0.942 | Pearson: 0.048<br>P=0.422 | Pearson: -0.041<br>P=0.492 | Pearson: -0.070<br>P=0.244 | Pearson: 0.077<br>P=0.198 |
| IL-17A | Pearson: -0.109<br>P=0.068 | Pearson: 0.065<br>P=0.281 | Pearson: -0.022<br>P=0.710 | Pearson: 0.019<br>P=0.752 | Pearson: 0.178<br>P=0.003 | Pearson: -0.026<br>P=0.660 | Pearson: 0.059<br>P=0.322 | Pearson: -0.020<br>P=0.741 | Pearson: -0.063<br>P=0.294 | Pearson: 0.016<br>P=0.791 |
| IP-10 | Pearson: -0.118<br>P=0.049 | Pearson: 0.031<br>P=0.609 | Pearson: 0.088<br>P=0.141 | Pearson: -0.031<br>P=0.610 | Pearson: 0.080<br>P=0.183 | Pearson: 0.026<br>P=0.667 | Pearson: 0.090<br>P=0.132 | Pearson: -0.091<br>P=0.128 | Pearson: -0.050<br>P=0.408 | Pearson: 0.030<br>P=0.618 |

| | | | | | | | | | | |
|---|---|---|---|---|---|---|---|---|---|---|
| MCP-1 | Pearson: -0.075 P=0.214 | Pearson: 0.014 P=0.810 | Pearson: 0.068 P=0.258 | Pearson: 0.027 P=0.653 | Pearson: 0.043 P=0.471 | Pearson: 0.064 P=0.284 | Pearson: 0.008 P=0.895 | Pearson: -0.050 P=0.404 | Pearson: -0.052 P=0.386 | Pearson: 0.010 P=0.865 |
| MIP-1A | Pearson: -0.010 P=0.864 | Pearson: 0.014 P=0.811 | Pearson: -0.027 P=0.658 | Pearson: -0.041 P=0.492 | Pearson: 0.103 P=0.085 | Pearson: -0.068 P=0.260 | Pearson: 0.031 P=0.601 | Pearson: 0.033 P=0.588 | Pearson: -0.023 P=0.700 | Pearson: -0.011 P=0.861 |
| MIP-1B | Pearson: -0.012 P=0.837 | Pearson: 0.054 P=0.371 | Pearson: -0.036 P=0.551 | Pearson: -0.023 P=0.696 | Pearson: -0.007 P=0.905 | Pearson: -0.053 P=0.378 | Pearson: -0.046 P=0.441 | Pearson: 0.084 P=0.161 | Pearson: -0.018 P=0.763 | Pearson: -0.002 P=0.972 |
| TNF-$\alpha$ | Pearson: -0.089 P=0.137 | Pearson: 0.042 P=0.484 | Pearson: -0.023 P=0.705 | Pearson: -0.025 P=0.677 | Pearson: 0.235 P=0.000 | Pearson: -0.055 P=0.361 | Pearson: 0.061 P=0.309 | Pearson: -0.005 P=0.929 | Pearson: -0.015 P=0.807 | Pearson: 0.002 P=0.968 |

CHIKV: chikungunya virus; Green: strong correlation; Yellow: moderate correlation; Red: weak correlation

**Supplementary Table S4.** Cramer's correlation analysis of demographics in patients with confirmed CHIKV infection.

| | Mestizo | Caucasian | Afro-American | Indigenous | Other | Illiteracy | Some education | Primary school | Middle school | High school | Bachelor | University | Strata 1 | Strata 2 | Strata 3 | Strata 4 | Strata 5 | Taxpayer | Beneficiary | Subsidized | Special regime | Private health care |
|---|---|---|---|---|---|---|---|---|---|---|---|---|---|---|---|---|---|---|---|---|---|---|
| Mestizo | | | | | | Cramer's: 0.05 p=0.34 OR: 1.6 (0.5-4.3) | Cramer's: 0.06 p=0.25 OR: 0.5 (0.2-1.4) | Cramer's: 0.03 p=0.52 OR: 1.1 (0.7-1.9) | Cramer's: 0.02 p=0.62 OR: 1.1 (0.6-2.2) | Cramer's: 0.07 p=0.23 OR: 0.7 (0.4-1.2) | Cramer's: 0.05 p=0.38 OR: 0.7 (0.3-1.4) | Cramer's: 0.01 p=0.80 OR: 0.8 (0.3-2.2) | Cramer's: 0.10 p=0.08 OR: 0.6 (0.4-1.1) | Cramer's: 0.17 p=0.003 OR: 2.1 (1.2-3.4) | Cramer's: 0.00 p=0.95 OR: 0.9 (0.5-1.7) | Cramer's: 0.05 p=0.38 OR: 0.5 (0.1-2.1) | Cramer's: 0.15 p=0.01 OR: 0.5 (0.4-0.5) | Cramer's: 0.00 p=0.93 OR: 0.9 (0.5-1.7) | Cramer's: 0.01 p=0.75 OR: 0.9 (0.5-1.6) | Cramer's: 0.02 p=0.66 OR: 1.1 (0.6-1.7) | Cramer's: 0.00 p=0.94 OR: 1.0 (0.6-17.7) | Cramer's: 0.05 p=0.33 OR: 0.52 (0.4-0.5) |
| Caucasian | | | | | | Cramer's: 0.07 p=0.20 OR: 0.4 (0.1-1.5) | Cramer's: 0.17 p=0.77 OR: 1.1 (0.4-2.9) | Cramer's: 0.04 p=0.49 OR: 0.8 (0.4-1.4) | Cramer's: 0.00 p=0.88 OR: 1.05 (0.5-2.0) | Cramer's: 0.07 p=0.19 OR: 1.4 (0.8-2.4) | Cramer's: 0.03 p=0.58 OR: 0.82 (0.4-1.6) | Cramer's: 0.01 p=0.84 OR: 1.09 (0.4-2.7) | Cramer's: 0.025 p=0.65 OR: 0.89 (0.5-1.4) | Cramer's: 0.08 p=0.14 OR: 0.68 (0.4-1.1) | Cramer's: 0.02 p=0.68 OR: 1.13 (0.6-2.0) | Cramer's: 0.06 p= 0.27 OR: 2.09 (0.5-7.9) | Cramer's: 0.20 p=0.001 OR: 0.36 (0.3-0.4) | Cramer's: 0.09 p=0.12 OR: 1.59 (0.8-2.9) | Cramer's: 0.00 p=0.95 OR: 0.98 (0.5-1.7) | Cramer's: 0.08 p=0.16 OR: 0.70 (0.4-1.1) | Cramer's: 0.02 p=0.72 OR: 1.63 (0.1-26.4) | Cramer's: 0.07 p=0.20 OR: 0.37 (0.3-0.4) |
| Afro-American | | | | | | Cramer's: 0.01 p=0.84 OR: 1.1 (0.2-5.3) | Cramer's: 0.04 p=0.41 OR: 2.3 (0.2-17.8) | Cramer's: 0.01 p=0.78 OR: 0.8 (0.3-2.0) | Cramer's: 0.00 p=0.88 OR: 0.9 (0.3-2.8) | Cramer's: 0.01 p=0.81 OR: 1.1 (0.4-2.6) | Cramer's: 0.05 p=0.33 OR: 1.6 (0.6-4.2) | Cramer's: 0.00 p=0.95 OR: 0.9 (0.2-4.3) | Cramer's: 0.16 p=0.006 OR: 2.9 | Cramer's: 0.10 p=0.07 OR: 0.44 | Cramer's: 0.02 p=0.65 OR: 0.79 | Cramer's: 0.06 p=0.29 OR: 0.89 | Cramer's: 0.05 p=0.36 OR: 0.89 | Cramer's: 0.10 p=0.07 OR: 0.28 | Cramer's: 0.01 p=0.87 OR: 0.9 (0.3-2.3) | Cramer's: 0.09 p=0.09 OR: 2.0 (0.8-4.7) | Cramer's: 0.02 p=0.62 OR: 0.8 (0.8-0.9) | Cramer's: 0.02 p=0.73 OR: 0.8 (0.8-0.9) |

| | | | | | | | | | | | | | | | | | | | | | | |
|---|---|---|---|---|---|---|---|---|---|---|---|---|---|---|---|---|---|---|---|---|---|---|
| | | | | | | | | | | | | | (1.3-46.5) | (0.1-1.1) | (0.2-2.1) | (0.8-0.9) | (0.8-0.9) | (0.0-1.2) | | | | |
| Indigenous | | | | | | Cramer’s: 0.04 p=0.46 OR: 0.96 (0.9-0.9) | Cramer’s: 0.04 p=0.42 OR: 1.0 (1.0-1.0) | Cramer’s: 0.07 p=0.20 OR: 2.4 (0.5-9.9) | Cramer’s: 0.07 p=0.23 OR: 0.96 (0.9-0.9) | Cramer’s: 0.00 p=0.95 OR: 0.95 (0.1-4.8) | Cramer’s: 0.05 p=0.40 OR: 1.98 (0.3-10.1) | Cramer’s: 0.04 p=0.42 OR: 0.96 (0.9-0.9) | Cramer’s: 0.00 p=0.96 OR: 0.96 (0.2-4.1) | Cramer’s: 0.00 p=0.38 OR: 0.52 (0.1-3.0) | Cramer’s: 0.01 p=0.74 OR: 1.3 (0.1-3.0) | Cramer’s: 0.09 p=0.13 OR: 4.6 (0.5-42.8) | Cramer’s: 0.02 p=0.64 OR: 0.9 (0.9-0.9) | Cramer’s: 0.02 p=0.63 OR: 0.60 (0.0-4.9) | Cramer’s: 0.06 p=0.27 OR: 2.2 (0.5-9.4) | Cramer’s: 0.28 p=0.63 OR: 0.7 (0.1-2.9) | Cramer’s: 0.05 p=0.80 OR: 0.9 (0.9-0.9) | Cramer’s: 0.01 p=0.86 OR: 0.9 (0.9-0.9) |
| Other | | | | | | Cramer’s: 0.11 p=0.04 OR: 8.1 (0.6-94.4) | Cramer’s: 0.02 p=0.62 OR: 1.0 (0.9-1.0) | Cramer’s: 0.06 p=0.25 OR: 0.9 (0.9-1.0) | Cramer’s: 0.04 p=0.47 OR: 0.9 (0.9-1.0) | Cramer’s: 0.06 p=0.30 OR: 0.9 (0.9-1.0) | Cramer’s: 0.15 p=0.01 OR: 12.1 (1-137.2) | Cramer’s: 0.10 p=0.07 OR: 6.7 (0.5-78.0) | Cramer’s: 0.13 p=0.02 OR: 1.0 (0.9-1.0) | Cramer’s: 0.07 p=0.19 OR: 0.9 (0.9-1.0) | Cramer’s: 0.05 p=0.37 OR: 0.9 (0.9-1.0) | Cramer’s: 0.01 p=0.75 OR: 0.9 (0.9-1.0) | Cramer’s: 0.01 p=0.78 OR: 0.9 (0.9-1.0) | Cramer’s: 0.05 p=0.39 OR: 0.9 (0.9-1.0) | Cramer’s: 0.02 p=0.62 OR: 1.8 (0.1-20.1) | Cramer’s: 0.01 p=0.76 OR: 1.4 (0.1-16.1) | Cramer’s: 0.00 p=0.88 OR: 0.9 (0.9-1.0) | Cramer’s: 0.00 p=0.91 OR: 0.9 (0.9-1.0) |
| Illiteracy | Cramer’s: 0.05 p=0.34 OR: 1.6 (0.5-4.3) | Cramer’s: 0.07 p=0.20 OR: 0.4 (0.1-1.5) | Cramer’s: 0.01 p=0.84 OR: 1.1 (0.2-5.3) | Cramer’s: 0.04 p=0.46 OR: 0.96 (0.9-0.9) | Cramer’s: 0.11 p=0.04 OR: 8.1 (0.6-94.4) | | | | | | | | Cramer’s: 0.16 p=0.00 OR: 4.2 (1.4-12.3) | Cramer’s: 0.06 p=0.28 OR: 0.5 (0.1-1.7) | Cramer’s: 0.09 p=0.12 OR: 0.2 (0.0-1.7) | Cramer’s: 0.04 p=0.43 OR: 0.9 (0.9-0.9) | Cramer’s: 0.04 p=0.49 OR: 0.9 (0.9-0.9) | Cramer’: 0.08 p=0.15 OR: 0.2 (0.0-1.9) | Cramer’: 0.13 p=0.02 OR: 0.9 (0.8-0.9) | Cramer’: 0.18 p=0.00 OR: 12.7 (1.6-97.2) | Cramer’: 0.02 p=0.71 OR: 0.9 (0.9-0.9) | Cramer’: 0.01 p=0.79 OR: 0.9 (0.9-0.9) |
| Some education | Cramer’s: 0.06 p=0.25 OR: 0.5 (0.2-1.4) | Cramer’s: 0.17 p=0.77 OR: 1.1 (0.4-2.9) | Cramer’s: 0.04 p=0.41 OR: 2.3 (0.2-17.8) | Cramer’s: 0.04 p=0.42 OR: 1.0 (1.0-1.0) | Cramer’s: 0.02 p=0.62 OR: 1.0 (0.9-1.0) | | | | | | | | Cramer’: 0.09 p=0.11 OR: 0.4 (0.1-1.2) | Cramer’: 0.00 p=0.96 OR: 1.0 (0.3-2.6) | Cramer’: 0.07 p=0.23 OR: 2.4 (0.5-10.7) | Cramer’: 0.05 p=0.39 OR: 0.9 (0.8-0.9) | Cramer’: 0.04 p=0.45 OR: 0.9 (0.8-0.9) | Cramer’: 0.00 p=0.90 OR: 0.9 (0.2-2.9) | Cramer’: 0.14 p=0.01 OR: 0.9 (0.8-0.9) | Cramer’: 0.12 p=0.03 OR: 0.3 (0.1-0.9) | Cramer’: 0.02 p=0.69 OR: 0.9 (0.8-0.9) | Cramer’: 0.01 p=0.78 OR: 0.9 (0.8-0.9) |
| Primary school | Cramer’s: 0.03 p=0.52 OR: 1.1 (0.7-1.9) | Cramer’s: 0.04 p=0.49 OR: 0.8 (0.4-1.4) | Cramer’s: 0.01 p=0.78 OR: 0.8 (0.3-2.0) | Cramer’s: 0.07 p=0.20 OR: 2.4 (0.5-9.9) | Cramer’s: 0.06 p=0.25 OR: 0.9 (0.9-1.0) | | | | | | | | Cramer’: 0.03 p=0.62 OR: 0.8 (0.5-1.4) | Cramer’: 0.01 p=0.07 OR: 1.6 (0.9-2.7) | Cramer’: 0.00 p=0.98 OR: 1.0 (0.5-1.8) | Cramer’: 0.11 p=0.04 OR: 0.6 (0.6-0.7) | Cramer’: 0.10 p=0.08 OR: 0.6 (0.6-0.7) | Cramer’: 0.07 p=0.20 OR: 0.6 (0.3-1.2) | Cramer’: 0.00 p=0.96 OR: 0.9 (0.5-1.8) | Cramer’: 0.07 p=0.20 OR: 1.4 (0.8-2.3) | Cramer’: 0.05 p=0.35 OR: 0.7 (0.6-0.7) | Cramer’: 0.03 p=0.51 OR: 0.7 (0.6-0.7) |
| Middle school | Cramer’s: 0.02 p=0.62 OR: 1.1 (0.6-2.2) | Cramer’s: 0.00 p=0.88 OR: 1.05 (0.5-2.0) | Cramer’s: 0.00 p=0.88 OR: 0.9 (0.3-2.8) | Cramer’s: 0.07 p=0.23 OR: 0.96 (0.9-0.9) | Cramer’s: 0.04 p=0.47 OR: 0.9 (0.9-1.0) | | | | | | | | Cramer’: 0.09 p=0.10 OR: 0.5 (0.2-1.1) | Cramer’: 0.05 p=0.38 OR: 1.3 (0.6-2.3) | Cramer’: 0.09 p=0.12 OR: 1.7 (0.8-3.7) | Cramer’: 0.01 p=0.75 OR: 0.7 (0.0-5.9) | Cramer’: 0.06 p=0.26 OR: 0.8 (0.8-0.8) | Cramer’: 0.00 p=0.92 OR: 1.0 (0.4-2.4) | Cramer’: 0.05 p=0.40 OR: 1.3 (0.6-2.9) | Cramer’: 0.03 p=0.53 OR: 0.8 (0.4-1.5) | Cramer’: 0.03 p=0.55 OR: 0.8 (0.8-0.8) | Cramer’: 0.02 p=0.67 OR: 0.8 (0.8-0.8) |

| | | | | | | | | | | | | | | | | | | | | | | |
|---|---|---|---|---|---|---|---|---|---|---|---|---|---|---|---|---|---|---|---|---|---|---|
| High school | Crame r’s: 0.07 p=0.23 OR: 0.7 (0.4- 1.2) | Cramer ’s: 0.07 p=0.19 OR: 1.4 (0.8- 2.4) | Crame r’s: 0.01 p=0.81 OR: 1.1 (0.4- 2.6) | Cramer’ s: 0.00 p=0.95 OR: 0.95 (0.1-4.8) | Crame r’s: 0.06 p=0.30 OR: 0.9 (0.9- 1.0) | | | | | | | | Crame r’: 0.05 p=0.34 OR: 1.3 (0.7- 2.2) | Crame r’: 0.02 p=0.65 OR: 0.8 (0.5- 1.5) | Crame r’: 0.11 p=0.05 OR: 0.4 (0.2- 1.0) | Crame r’: 0.12 p=0.03 OR: 3.7 (0.9- 14.5) | Crame r’: 0.06 p=0.29 OR: 2.2 (0.4- 10.1) | Crame r’: 0.01 p=0.81 OR: 0.9 (0.4- 1.8) | Cramer’ : 0.00 p=0.93 OR: 0.1 (0.5-1.9) | Cramer’ : 0.00 p=0.95 OR: 1.0 (0.5-1.7) | Crame r’: 0.04 p=0.43 OR: 2.9 (0.1- 46.9) | Crame r’: 0.03 p=0.55 OR: 0.7 (0.6- 0.7) |
| Bachelo r | Crame r’s: 0.05 p=0.38 OR: 0.7 (0.3- 1.4) | Cramer ’s: 0.03 p=0.58 OR: 0.82 (0.4- 1.6) | Crame r’s: 0.05 p=0.33 OR: 1.6 (0.6- 4.2) | Cramer’ s: 0.05 p=0.40 OR: 1.98 (0.3- 10.1) | Crame r’s: 0.15 p=0.01 OR: 12.1 (1- 137.2) | | | | | | | | Crame r’: 0.04 p=0.42 OR: 1.3 (0.6- 2.5) | Crame r’: 0.11 p=0.05 OR: 0.4 (0.2- 1.0) | Crame r’: 0.06 p=0.27 OR: 0.15 (0.7- 3.2) | Crame r’: 0.01 p=0.75 OR: 0.07 (0.0- 5.9) | Crame r’: 0.06 p=0.29 OR: 0.23 (0.4- 12.7) | Crame r’: 0.00 p=0.92 OR: 1.0 (0.4- 2.4) | Cramer’ : 0.07 p=0.21 OR: 1.5 (0.7-3.3) | Cramer’ : 0.07 p=0.19 OR: 0.6 (0.3-1.2) | Crame r’: 0.03 p=0.55 OR: 0.8 (0.8- 0.8) | Crame r’: 0.14 p=0.01 OR: 0.1 (0.1- 0.1) |
| Universi ty | Crame r’s: 0.01 p=0.80 OR: 0.8 (0.3- 2.2) | Cramer ’s: 0.01 p=0.84 OR: 1.09 (0.4- 2.7) | Crame r’s: 0.00 p=0.95 OR: 0.9 (0.2- 4.3) | Cramer’ s: 0.04 p=0.42 OR: 0.96 (0.9-0.9) | Crame r’s: 0.10 p=0.07 OR: 6.7 (0.5- 78.0) | | | | | | | | Crame r’: 0.04 p=0.42 OR: 0.6 (0.2- 1.8) | Crame r’: 0.06 p=0.30 OR: 0.5 (0.2- 1.6) | Crame r’: 0.03 p=0.59 OR: 1.3 (0.4- 3.8) | Crame r’: 0.10 p=0.07 OR: 4.0 (0.7- 20.6) | Crame r’: 0.13 p=0.02 OR: 5.6 (1.0- 31.1) | Crame r’: 0.07 p=0.19 OR: 1.9 (0.7- 5.2) | Cramer’ : 0.01 p=0.83 OR: 0.8 (0.2-2.7) | Cramer’ : 0.07 p=0.21 OR: 0.5 (0.2-1.4) | Crame r’: 0.14 p=0.01 OR: 13.5 (0.8- 225) | Crame r’: 0.01 p=0.78 OR: 0.9 (0.8- 0.9) |
| Strata 1 | Crame r’s: 0.10 p=0.08 OR: 0.6 (0.4- 1.1) | Cramer ’s: 0.025 p=0.65 OR: 0.89 (0.5- 1.4) | Crame r’s: 0.16 p=0.00 6 OR: 2.9 (1.3- 46.5) | Cramer’ s: 0.00 p=0.96 OR: 0.96 (0.2-4.1) | Crame r’s: 0.13 p=0.02 OR: 1.0 (0.9- 1.0) | Crame r’s: 0.16 p=0.00 OR: 4.2 (1.4- 12.3) | Crame r’: 0.09 p=0.11 OR: 0.4 (0.1- 1.2) | Crame r’: 0.03 p=0.62 OR: 0.8 (0.5- 1.4) | Crame r’: 0.09 p=0.10 OR: 0.5 (0.2- 1.1) | Crame r’: 0.05 p=0.34 OR: 1.3 (0.7- 2.2) | Crame r’: 0.04 p=0.42 OR: 1.3 (0.6- 2.5) | Cramer ’: 0.04 p=0.42 OR: 0.6 (0.2-1.8) | | | | | | Crame r’: 0.15 p=0.00 OR: 0.4 (0.2- 0.8) | Cramer’ : 0.13 p=0.02 OR: 0.4 (0.2-0.9) | Cramer’ : 0.23 p=0.00 OR: 2.8 (1.6-4.7) | Crame r’: 0.02 p=0.73 OR: 1.6 (0.1-26) | Crame r’: 0.04 p=0.42 OR: 0.6 (0.5- 0.6) |
| Strata 2 | Crame r’s: 0.17 p=0.00 3 OR: 2.1 (1.2- 3.4) | Cramer ’s: 0.08 p=0.14 OR: 0.68 (0.4- 1.1) | Crame r’s: 0.10 p=0.07 OR: 0.44 (0.1- 1.1) | Cramer’ s: 0.03 p=0.52 OR: 0.59 (0.1-3.0) | Crame r’s: 0.07 p=0.19 OR: 0.9 (0.9- 1.0) | Crame r’s: 0.06 p=0.28 OR: 0.5 (0.1- 1.7) | Crame r’: 0.00 p=0.96 OR: 1.0 (0.3- 2.6) | Crame r’: 0.01 p=0.07 OR: 1.6 (0.9- 2.7) | Crame r’: 0.05 p=0.38 OR: 1.3 (0.6- 2.3) | Crame r’: 0.02 p=0.65 OR: 0.8 (0.5- 1.5) | Crame r’: 0.11 p=0.05 OR: 0.4 (0.2- 1.0) | Cramer ’: 0.06 p=0.30 OR: 0.5 (0.2-1.6) | | | | | | Crame r’: 0.06 p=0.30 OR: 1.3 (0.7- 2.5) | Cramer’ : 0.07 p=0.18 OR: 1.4 (0.8-2.6) | Cramer’ : 0.09 p=0.10 OR: 0.6 (0.4-1.0) | Crame r’: 0.06 p=0.29 OR: 0.6 (0.5- 0.7) | Crame r’: 0.04 p=0.45 OR: 0.6 (0.5- 0.7) |
| Strata 3 | Crame r’s: 0.00 p=0.95 OR: 0.9 (0.5- 1.7) | Cramer ’s: 0.02 p=0.68 OR: 1.13 (0.6- 2.0) | Crame r’s: 0.02 p=0.65 OR: 0.79 (0.2- 2.1) | Cramer’ s: 0.01 p=0.74 OR: 1.3 (0.1-3.0) | Crame r’s: 0.05 p=0.37 OR: 0.9 (0.9- 1.0) | Crame r’s: 0.09 p=0.12 OR: 0.2 (0.0- 1.7) | Crame r’: 0.07 p=0.23 OR: 2.4 (0.5- 10.7) | Crame r’: 0.00 p=0.98 OR: 1.0 (0.5- 1.8) | Crame r’: 0.09 p=0.12 OR: 1.7 (0.8- 3.7) | Crame r’: 0.11 p=0.05 OR: 0.4 (0.2- 1.0) | Crame r’: 0.06 p=0.27 OR: 0.15 (0.7- 3.2) | Cramer ’: 0.03 p=0.59 OR: 1.3 (0.4-3.8) | | | | | | Crame r’: 0.11 p=0.05 OR: 1.9 (0.9- 03.8) | Cramer’ : 0.03 p=0.58 OR: 1.2 (0.6-2.4) | Cramer’ : 0.12 p=0.03 OR: 0.5 (0.2-0.9) | Crame r’: 0.06 p=0.29 OR: 3.9 (0.2- 64.0) | Crame r’: 0.03 p=0.61 OR: 0.7 (0.7- 0.8) |

| | | | | | | | | | | | |
|---|---|---|---|---|---|---|---|---|---|---|---|
| Strata 4 | Cramer's: 0.05 p=0.38 OR: 0.5 (0.1-2.1) | Cramer's: 0.06 p= 0.27 OR: 2.09 (0.5-7.9) | Cramer's: 0.06 p=0.29 OR: 0.89 (0.8-0.9) | Cramer′s: 0.09 p=0.13 OR: 4.6 (0.5-42.8) | Cramer's: 0.01 p=0.75 OR: 0.9 (0.9-1.0) | Cramer's: 0.04 p=0.43 OR: 0.9 (0.9-0.9) | Cramer′: 0.05 p=0.39 OR: 0.9 (0.8-0.9) | Cramer′: 0.11 p=0.04 OR: 0.6 (0.6-0.7) | Cramer′: 0.01 p=0.75 OR: 0.7 (0.0-5.9) | Cramer′: 0.12 p=0.03 OR: 3.7 (0.9-14.5) | Cramer′: 0.01 p=0.75 OR: 0.07 (0.0-5.9) |
| Strata 5 | Cramer's: 0.15 p=0.01 OR: 0.5 (0.4-0.5) | Cramer's: 0.20 p=0.001 OR: 0.36 (0.3-0.4) | Cramer's: 0.05 p=0.36 OR: 0.89 (0.8-0.9) | Cramer′s: 0.02 p=0.64 OR: 0.9 (0.9-0.9) | Cramer's: 0.01 p=0.78 OR: 0.9 (0.9-1.0) | Cramer's: 0.04 p=0.49 OR: 0.9 (0.9-0.9) | Cramer′: 0.04 p=0.45 OR: 0.9 (0.8-0.9) | Cramer′: 0.10 p=0.08 OR: 0.6 (0.6-0.7) | Cramer′: 0.06 p=0.26 OR: 0.8 (0.8-0.8) | Cramer′: 0.06 p=0.29 OR: 2.2 (0.4-10.1) | Cramer′: 0.06 p=0.29 OR: 0.23 (0.4-12.7) |
| Taxpayer | Cramer's: 0.00 p=0.93 OR: 0.9 (0.5-1.7) | Cramer's: 0.09 p=0.12 OR: 1.59 (0.8-2.9) | Cramer's: 0.10 p=0.07 OR: 0.28 (0.0-1.2) | Cramer′s: 0.02 p=0.63 OR: 0.60 (0.0-4.9) | Cramer's: 0.05 p=0.39 OR: 0.9 (0.9-1.0) | Cramer′: 0.08 p=0.15 OR: 0.2 (0.0-1.9) | Cramer′: 0.00 p=0.90 OR: 0.9 (0.2-2.9) | Cramer′: 0.07 p=0.20 OR: 0.6 (0.3-1.2) | Cramer′: 0.00 p=0.92 OR: 1.0 (0.4-2.4) | Cramer′: 0.01 p=0.81 OR: 0.9 (0.4-1.8) | Cramer′: 0.00 p=0.92 OR: 1.0 (0.4-2.4) |
| Beneficiary | Cramer's: 0.01 p=0.75 OR: 0.9 (0.5-1.6) | Cramer's: 0.00 p=0.95 OR: 0.98 (0.5-1.7) | Cramer's: 0.01 p=0.87 OR: 0.9 (0.3-2.3) | Cramer′s: 0.06 p=0.27 OR: 2.2 (0.5-9.4) | Cramer's: 0.02 p=0.62 OR: 1.8 (0.1-20.1) | Cramer′: 0.13 p=0.02 OR: 0.9 (0.8-0.9) | Cramer′: 0.14 p=0.01 OR: 0.9 (0.8-0.9) | Cramer′: 0.00 p=0.96 OR: 0.9 (0.5-1.8) | Cramer′: 0.05 p=0.40 OR: 1.3 (0.6-2.9) | Cramer′: 0.00 p=0.93 OR: 0.1 (0.5-1.9) | Cramer′: 0.07 p=0.21 OR: 1.5 (0.7-3.3) |
| Subsidized | Cramer's: 0.02 p=0.66 OR: 1.1 (0.6-1.7) | Cramer's: 0.08 p=0.16 OR: 0.70 (0.4-1.1) | Cramer's: 0.09 p=0.09 OR: 2.0 (0.8-4.7) | Cramer′s: 0.28 p=0.63 OR: 0.7 (0.1-2.9) | Cramer's: 0.01 p=0.76 OR: 1.4 (0.1-16.1) | Cramer′: 0.18 p=0.00 OR: 12.7 (1.6-97.2) | Cramer′: 0.12 p=0.03 OR: 0.3 (0.1-0.9) | Cramer′: 0.07 p=0.20 OR: 1.4 (0.8-2.3) | Cramer′: 0.03 p=0.53 OR: 0.8 (0.4-1.5) | Cramer′: 0.00 p=0.95 OR: 1.0 (0.5-1.7) | Cramer′: 0.07 p=0.19 OR: 0.6 (0.3-1.2) |
| Special regime | Cramer's: 0.00 p=0.94 OR: 1.0 (0.6-17.7) | Cramer's: 0.02 p=0.72 OR: 1.63 (0.1-26.4) | Cramer's: 0.02 p=0.62 OR: 0.8 (0.8-0.9) | Cramer′s: 0.05 p=0.80 OR: 0.9 (0.9-0.9) | Cramer's: 0.00 p=0.88 OR: 0.9 (0.9-1.0) | Cramer′: 0.02 p=0.71 OR: 0.9 (0.9-0.9) | Cramer′: 0.02 p=0.69 OR: 0.9 (0.8-0.9) | Cramer′: 0.05 p=0.35 OR: 0.7 (0.6-0.7) | Cramer′: 0.03 p=0.55 OR: 0.8 (0.8-0.8) | Cramer′: 0.04 p=0.43 OR: 2.9 (0.1-46.9) | Cramer′: 0.03 p=0.55 OR: 0.8 (0.8-0.8) |

| | | | | | | | | | | | |
|---|---|---|---|---|---|---|---|---|---|---|---|
| Strata 4 | Cramer′: 0.10 p=0.07 OR: 4.0 (0.7-20.6) | | | | | | Cramer′: 0.01 p=0.80 OR: 1.2 (0.2-6.0) | Cramer′: 0.00 p=0.97 OR: 1.0 (0.2-5.0) | Cramer′: 0.00 p=0.87 OR: 0.9 (0.2-3.4) | Cramer′: 0.01 p=0.79 OR: 0.9 (0.9-0.9) | Cramer′: 0.01 p=0.85 OR: 0.9 (0.9-0.9) |
| Strata 5 | Cramer′: 0.13 p=0.02 OR: 5.6 (1.0-31.1) | | | | | | Cramer′: 0.01 p=0.74 OR: 0.7 (0.0-5.9) | Cramer′: 0.08 p=0.17 OR: 2.7 (0.6-12.7) | Cramer′: 0.09 p=0.10 OR: 0.2 (0.0-1.4) | Cramer′: 0.01 p=0.82 OR: 0.9 (0.9-0.9) | Cramer′: 0.37 p=0.00 OR: 0.02 (0.0-0.0) |
| Taxpayer | Cramer′: 0.07 p=0.19 OR: 1.9 (0.7-5.2) | Cramer′: 0.15 p=0.00 OR: 0.4 (0.2-0.8) | Cramer′: 0.06 p=0.30 OR: 1.3 (0.7-2.5) | Cramer′: 0.11 p=0.05 OR: 1.9 (0.9-03.8) | Cramer′: 0.01 p=0.80 OR: 1.2 (0.2-6.0) | Cramer′: 0.01 p=0.74 OR: 0.7 (0.0-5.9) | | | | | |
| Beneficiary | Cramer′: 0.01 p=0.83 OR: 0.8 (0.2-2.7) | Cramer′: 0.13 p=0.02 OR: 0.4 (0.2-0.9) | Cramer′: 0.07 p=0.18 OR: 1.4 (0.8-2.6) | Cramer′: 0.03 p=0.58 OR: 1.2 (0.6-2.4) | Cramer′: 0.00 p=0.97 OR: 1.0 (0.2-5.0) | Cramer′: 0.08 p=0.17 OR: 2.7 (0.6-12.7) | | | | | |
| Subsidized | Cramer′: 0.07 p=0.21 OR: 0.5 (0.2-1.4) | Cramer′: 0.23 p=0.00 OR: 2.8 (1.6-4.7) | Cramer′: 0.09 p=0.10 OR: 0.6 (0.4-1.0) | Cramer′: 0.12 p=0.03 OR: 0.5 (0.2-0.9) | Cramer′: 0.00 p=0.87 OR: 0.9 (0.2-3.4) | Cramer′: 0.09 p=0.10 OR: 0.2 (0.0-1.4) | | | | | |
| Special regime | Cramer′: 0.14 p=0.01 OR: 13.5 (0.8-225) | Cramer′: 0.02 p=0.73 OR: 1.6 (0.1-26) | Cramer′: 0.06 p=0.29 OR: 0.6 (0.5-0.7) | Cramer′: 0.06 p=0.29 OR: 3.9 (0.2-64.0) | Cramer′: 0.01 p=0.79 OR: 0.9 (0.9-0.9) | Cramer′: 0.01 p=0.82 OR: 0.9 (0.9-0.9) | | | | | |

| | | | | | | | | | | | | | | | | | |
|---|---|---|---|---|---|---|---|---|---|---|---|---|---|---|---|---|---|
| Private health care | Cramer’s: 0.05 p=0.33 OR: 0.52 (0.4-0.5) | Cramer’s: 0.07 p=0.20 OR: 0.37 (0.3-0.4) | Cramer’s: 0.02 p=0.73 OR: 0.8 (0.8-0.9) | Cramer’s: 0.01 p=0.86 OR: 0.9 (0.9-0.9) | Cramer’s: 0.00 p=0.91 OR: 0.9 (0.9-1.0) | Cramer’: 0.01 p=0.79 OR: 0.9 (0.9-0.9) | Cramer’: 0.01 p=0.78 OR: 0.9 (0.8-0.9) | Cramer’: 0.03 p=0.51 OR: 0.7 (0.6-0.7) | Cramer’: 0.02 p=0.67 OR: 0.8 (0.8-0.8) | Cramer’: 0.03 p=0.55 OR: 0.7 (0.6-0.7) | Cramer’: 0.14 p=0.01 OR: 0.1 (0.1-0.1) | Cramer’: 0.01 p=0.78 OR: 0.9 (0.8-0.9) | Cramer’: 0.04 p=0.42 OR: 0.6 (0.5-0.6) | Cramer’: 0.04 p=0.45 OR: 0.6 (0.5-0.7) | Cramer’: 0.03 p=0.61 OR: 0.7 (0.7-0.8) | Cramer’: 0.01 p=0.85 OR: 0.9 (0.9-0.9) | Cramer’: 0.37 p=0.00 OR: 0.02 (0.0-0.0) |

CHIKV: chikungunya virus; Green: strong correlation; Yellow: moderate correlation; Red: weak correlation

**Supplementary Table S5.** Cramer’s correlation analysis of demographics and clinical symptoms in patients with confirmed CHIKV infection.

| | Arthritis | Fever | Myalgia | Fatigue | Rash | G/I symptoms |
|---|---|---|---|---|---|---|
| Mestizo | Cramer’s: 0.01 P=0.865 OR: 1.0 (0.6-1.7) | Cramer’s: 0.03 P=0.595 OR: 0.8 (0.5-1.4) | Cramer’s: 0.01 P=0.807 OR: 0.9 (0.5-1.5) | Cramer’s: 0.04 P=0.433 OR: 0.8 (0.5-1.3) | Cramer’s: 0.01 P=0.798 OR: 0.9 (0.5-1.5) | Cramer’s: 0.09 P=0.099 OR: 0.6 (0.3-1,0) |
| Caucasian | Cramer’s: 0.02 P=0.727 OR: 0.9 (0.5-1.5) | Cramer’s: 0.06 P=0.274 OR: 1.3 (0.8-2.1) | Cramer’s: 0.00 P=0.927 OR: 1.0 (0.6-1.6) | Cramer’s: 0.06 P=0.266 OR: 1.3 (0.8-2.1) | Cramer’s: 0.03 P=0.598 OR: 1.1 (0.7-1.8) | Cramer’s: 0.121 P=0.043 OR: 1.7 (1.0-295) |
| Afro-American | Cramer’s: 0.00 P=0.890 OR: 1.0 (0.4-2.3) | Cramer’s: 0.06 P=0.297 OR: 0.6 (0.3-1.4) | Cramer’s: 0.05 P=0.364 OR: 0.6 (0.3-1.5) | Cramer’s: 0.04 P=0.465 OR: 0.7 (0.3-1.6) | Cramer’s: 0.02 P=0.665 OR: 0.8 (0.3-1.8) | Cramer’s: 0.00 P=0.963 OR: 0.9 (0.4-2.3) |
| Indigenous | Cramer’s: 0.07 P=0.205 OR: 0.2 (0.0-2.2) | Cramer’s: 0.04 P=0.454 OR: 0.5 (0.1-2.4) | Cramer’s: 0.05 P=0.338 OR: 2.0 (0.4-8.5) | Cramer’s: 0.02 P=0.639 OR: 0.7 (0.1-2.9) | Cramer’s: 0.02 P=0.659 OR: 0.7 (0.1-3.0) | Cramer’s: 0.01 P=0.850 OR: 0.8 (0.1-4.3) |
| Other | Cramer’s: 0.14 P=0.014 OR: 0.3 (0.2-0.3) | Cramer’s: 0.10 P=0.085 OR: 0.5 (0.4-0.5) | Cramer’s: 0.11 P=0.059 OR: 0.4 (0.3-0.5) | Cramer’s: 0.08 P=0.139 OR: 0.5 (0.5-0.6) | Cramer’s: 0.04 P=0.452 OR: 2.4 (0.2-27.3) | Cramer’s: 0.06 P=0.278 OR: 0.7 (0.6-0.7) |

| | | | | | | |
|---|---|---|---|---|---|---|
| Some education | Cramer's: 0.01<br>P=0.870<br>OR: 0.9 (0.3-2.3) | Cramer's: 0.03<br>P=0.607<br>OR: 1.2 (0.5-3.1) | Cramer's: 0.08<br>P=0.139<br>OR: 2.0 (0.7-5.5) | Cramer's: 0.01<br>P=0.773<br>OR: 1.1 (0.4-2.8) | Cramer's: 0.140<br>P=0.019<br>OR: 3.5 (1.1-10.9) | Cramer's: 0.01<br>P=0.833<br>OR: 0.8 (0.3-2.4) |
| Primary school | Cramer's: 0.02<br>P=0.643<br>OR: 0.8 (0.5-1.5) | Cramer's: 0.12<br>P=0.037<br>OR: 0.5 (0.3-0.9) | Cramer's: 0.01<br>P=0.777<br>OR: 0.9 (0.5-1.5) | Cramer's: 0.146<br>P=0.015<br>OR: 0.5 (0.3-0.8) | Cramer's: 0.03<br>P=0.513<br>OR: 0.8 (0.5-1.4) | Cramer's: 0.07<br>P=0.220<br>OR: 0.6 (0.3-1.2) |
| Middle school | Cramer's: 0.03<br>P=0.550<br>OR: 0.8 (0.3-1.6) | Cramer's: 0.00<br>P=0.925<br>OR: 1.0 (0.5-2.0) | Cramer's: 0.03<br>P=0.539<br>OR: 0.8 (0.4-1.5) | Cramer's: 0.01<br>P=0.782<br>OR: 0.9 (0.4-1.7) | Cramer's: 0.05<br>P=0.399<br>OR: 1.3 (0.6-2.5) | Cramer's: 0.01<br>P=0.862<br>OR: 0.9 (0.4-1.9) |
| High school | Cramer's: 0.08<br>P=0.147<br>OR: 0.6 (0.3-1.1) | Cramer's: 0.00<br>P=0.916<br>OR: 0.9 (0.5-1.6) | Cramer's: 0.03<br>P=0.589<br>OR: 1.1 (0.6-1.9) | Cramer's: 0.02<br>P=0.741<br>OR: 1.0 (0.6-1.8) | Cramer's: 0.00<br>P=0.887<br>OR: 0.9 (0.5-1.6) | Cramer's: 0.01<br>P=0.791<br>OR: 1.0 (0.5-1.9) |
| Bachelor | Cramer's: 0.09<br>P=0.120<br>OR: 1.7 (0.8-3.3) | Cramer's: 0.04<br>P=0.441<br>OR: 1.2 (0.6-2.5) | Cramer's: 0.02<br>P=0.686<br>OR: 1.1 (0.5-2.2) | Cramer's: 0.127<br>P=0.034<br>OR: 2.1 (1.0-4.5) | Cramer's: 0.07<br>P=0.237<br>OR: 1.4 (0.7-2.9) | Cramer's: 0.03<br>P=0.562<br>OR: 1.2 (0.6-2.5) |
| University | Cramer's: 0.12<br>P=0.033<br>OR: 2.6 (1.0-6.6) | Cramer's: 0.21<br>P=0.000<br>OR: 9.9 (2.2-43.7) | Cramer's: 0.107<br>P=0.075<br>OR: 2.3 (0.8-6.0) | Cramer's: 0.124<br>P=0.039<br>OR: 3.0 (1.0-9.5) | Cramer's: 0.08<br>P=0.166<br>OR: 1.9 (0.7-4.8) | Cramer's: 0.07<br>P=0.213<br>OR: 1.8 (0.7-4.5) |
| Strata 1 | Cramer's: 0.22<br>P=0.000<br>OR: 2.6 (1.5-4.3) | Cramer's: 0.176<br>P=0.003<br>OR: 2.0 (1.2-3.4) | Cramer's: 0.147<br>P=0.014<br>OR: 1.8 (1.1-2.9) | Cramer's: 0.252<br>P=0.000<br>OR: 3.0 (1.7-5.1) | Cramer's: 0.232<br>P=0.000<br>OR: 2.6 (1.6-4.3) | Cramer's: 0.215<br>P=0.000<br>OR: 2.6 (1.5-4.4) |
| Strata 2 | Cramer's: 0.09<br>P=0.111<br>OR: 0.6 (0.3-1.1) | Cramer's: 0.16<br>P=0.006<br>OR: 0.4 (0.3-0.8) | Cramer's: 0.217<br>P=0.000<br>OR: 0.3 (0.2-0.6) | Cramer's: 0.265<br>P=0.000<br>OR: 0.3 (0.1-0.5) | Cramer's: 0.176<br>P=0.003<br>OR: 0.4 (0.2-0.7) | Cramer's: 0.178<br>P=0.003<br>OR: 0.4 (0.2-0.7) |
| Strata 3 | Cramer's: 0.11<br>P=0.059 | Cramer's: 0.01<br>P=0.811 | Cramer's: 0.03<br>P=0.582 | Cramer's: 0.03<br>P=0.567 | Cramer's: 0.06<br>P=0.264 | Cramer's: 0.07<br>P=0.193 |

| | | | | | | |
|---|---|---|---|---|---|---|
| | OR: 0.5 (0.2-1.0) | OR: 0.9 (0.5-1.6) | OR: 1.1 (0.6-2.1) | OR: 1.1 (0.6-2.1) | OR: 0.7 (0.3-1.2) | OR: 0.6 (0.3-1.2) |
| Strata 4 | Cramer's: 0.12<br>P=0.031<br>OR: 0.6 (0.6-0.7) | Cramer's: 0.02<br>P=0.710<br>OR: 0.7 (0.2-2.9) | Cramer's: 0.04<br>P=0.443<br>OR: 0.5 (0.1-2.3) | Cramer's: 0.09<br>P=0.126<br>OR: 0.3 (0.0-1.4) | Cramer's: 0.04<br>P=0.469<br>OR: 0.5 (0.1-2.4) | Cramer's: 0.02<br>P=0.715<br>OR: 1.3 (0.3-5.3) |
| Strata 5 | Cramer's: 0.03<br>P=0.588<br>OR: 1.5 (0.3-6.9) | Cramer's: 0.02<br>P=0.723<br>OR: 1.3 (0.2-5.9) | Cramer's: 0.174<br>P=0.004<br>OR: 0.4 (0.3-0.5) | Cramer's: 0.04<br>P=0.468<br>OR: 1.8 (0.3-9.6) | Cramer's: 0.03<br>P=0.519<br>OR: 1.6 (0.3-7.4) | Cramer's: 0.05<br>P=0.374<br>OR: 1.9 (0.4-9.0) |
| Taxpayer | Cramer's: 0.11<br>P=0.067<br>OR: 0.5 (0.2-1.0) | Cramer's: 0.16<br>P=0.007<br>OR: 0.4 (0.2-0.8) | Cramer's: 0.22<br>P=0.000<br>OR: 0.2 (0.1-0.5) | Cramer's: 0.218<br>P=0.000<br>OR: 0.3 (0.1-0.6) | Cramer's: 0.201<br>P=0.001<br>OR: 0.3 (0.1-0.6) | Cramer's: 0.139<br>P=0.020<br>OR: 0.3 (0.1-0.8) |
| Beneficiary | Cramer's: 0.01<br>P=0.838<br>OR: 1.0 (0.5-1.9) | Cramer's: 0.00<br>P=0.960<br>OR: 1.0 (0.5-1.7) | Cramer's: 0.05<br>P=0.381<br>OR: 1.2 (0.7-2.2) | Cramer's: 0.04<br>P=0.478<br>OR: 0.8 (0.4-1.4) | Cramer's: 0.04<br>P=0.475<br>OR: 1.2 (0.6-2.1) | Cramer's: 0.04<br>P=0.507<br>OR: 0.8 (0.4-1.5) |
| Subsidized | Cramer's: 0.06<br>P=0.303<br>OR: 1.3 (0.7-2.1) | Cramer's: 0.11<br>P=0.049<br>OR: 1.6 (1.0-2.6) | Cramer's: 0.126<br>P=0.035<br>OR: 1.6 (1.0-2.7) | Cramer's: 0.205<br>P=0.001<br>OR: 2.3 (1.4-3.8) | Cramer's: 0.144<br>P=0.016<br>OR: 1.8 (1.1-2.9) | Cramer's: 0.141<br>P=0.019<br>OR: 1.9 (1.1-3.3) |
| Special regime | Cramer's: 0.03<br>P=0.616<br>OR: 2.0 (0.1-32.5) | Cramer's: 0.00<br>P=0.988<br>OR: 0.9 (0.0-15.8) | Cramer's: 0.07<br>P=0.907<br>OR: 1.1 (0.0-19.0) | Cramer's: 0.01<br>P=0.817<br>OR: 0.7 (0.0-11.6) | Cramer's: 0.07<br>P=0.198<br>OR: 0.5 (0.4-0.6) | Cramer's: 0.05<br>P=0.377<br>OR: 0.7 (0.6-0.7) |
| Private health care | Cramer's: 0.08<br>P=0.157<br>OR: 0.3 (0.2-0.3) | Cramer's: 0.05<br>P=0.322<br>OR: 0.5 (0.4-0.5) | Cramer's: 0.06<br>P=0.277<br>OR: 1.8 (1.1-3.0) | Cramer's: 0.05<br>P=0.395<br>OR: 0.5 (0.5-0.6) | Cramer's: 0.05<br>P=0.363<br>OR: 0.5 (0.4-0.6) | Cramer's: 0.09<br>P=0.108<br>OR: 0.2 (0.2-0.3) |

CHIKV: chikungunya virus; Green: strong correlation; Yellow: moderate correlation; Red: weak correlation

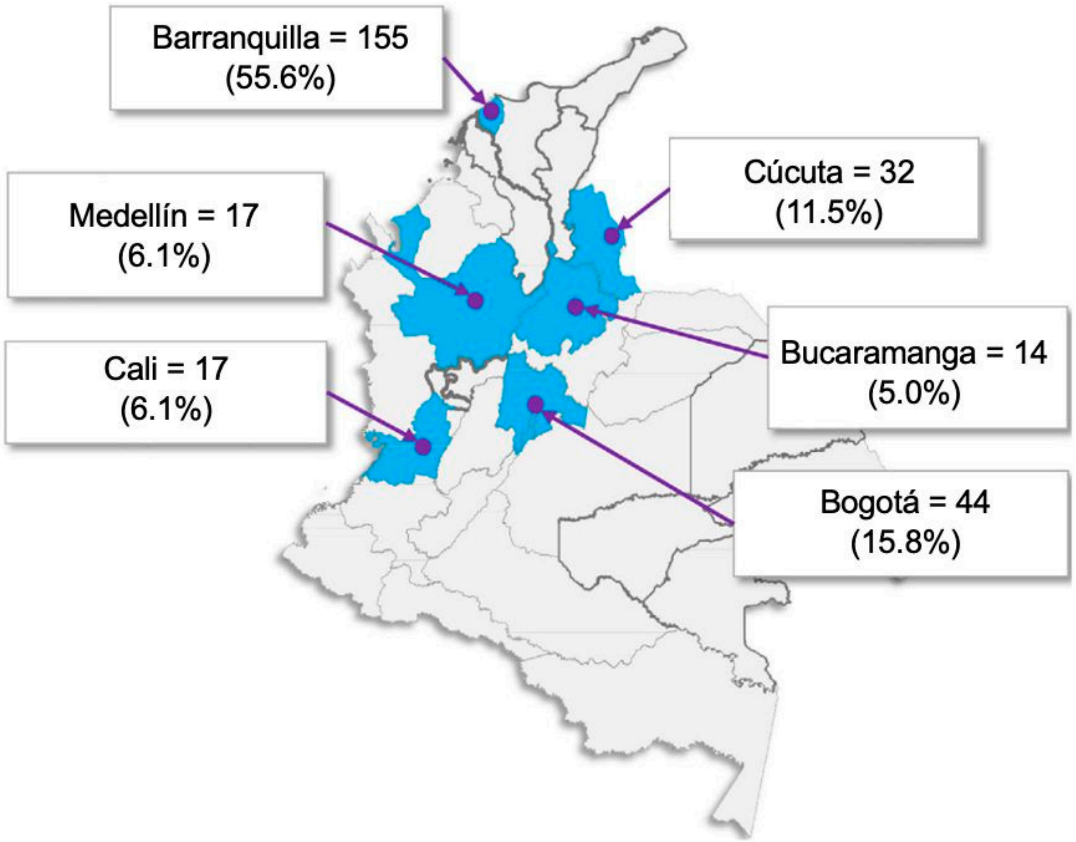


**Supplementary Figure S1.** Map of the cities studied that received a positive COPCORD score. COPCORD: Community Program for the Control of Rheumatic Diseases
